# Supplementary material for: Effectiveness and cost-effectiveness of personalised dietary advice aiming at increasing protein intake on physical functioning in community-dwelling older adults with lower habitual protein intake: rationale and design of the PROMISS randomised controlled trial
Source: BMJ Open. 2020 Nov 20;10(11):e040637. doi: 10.1136/bmjopen-2020-040637 (PMC7682452; doi:10.1136/bmjopen-2020-040637)
Supplement: Supplementary data [file bmjopen-2020-040637supp001.pdf]

## Appendix

## Appendix I

**<u>PROMISS project coordination, Vrije Universiteit Amsterdam, Department of Health Sciences, the Netherlands</u>**

Prof. Marjolein Visser, PhD – Principle investigator of the PROMISS project

Prof. Ingeborg A Brouwer, PhD – Project manager of the PROMISS project

Margreet Olthof, PhD – Financial manager of the PROMISS project

Rachel Vijlbrief – Assistant project manager of the PROMISS project

*Trial sites*

<u>Vrije Universiteit Amsterdam, the Netherlands</u>

Hanneke Wijnhoven, PhD - local principal investigator

Nanouk Bakker Schut – research intern

Judith Bosmans, PhD – researcher

Mariska Bout – dietician and research assistant

Ingeborg Brouwer, PhD – researcher

Nona Kerremans – research intern

Lothar Kuijper, PhD – researcher

Margreet Olthof, PhD – researcher

Ilse Reinders, PhD – local co-principal investigator

Marjon Veeke – dietician and research assistant

Rachel Vijlbrief – researcher

Marjolein Visser, PhD – researcher

Merel Vrijmoeth – dietician and research assistant

Laura Winkens – researcher

University of Helsinki

Merja Suominen, PhD – local principal investigator

Kirsi Ali-Kovero – research assistant

Johannes Anttila – research intern

Aliisa Hyvönen – dietician and research assistant

Henriikka Jussila – research intern

Satu Jyväkorpi, PhD – local co-principal investigator

Riikka Niskanen – dietician and research assistant

Anna-Maria Piipponen – research intern and research assistant

Kaisu Pitkälä, PhD – researcher

Heli Salmenius-Suominen – researcher

*Ancillary studies*

Persuasive technology study

Michel Klein, PhD – principal investigator of the persuasive technology study, *Vrije Universiteit Amsterdam, the Netherlands*

Laura van der Lubbe – researcher, *Vrije Universiteit Amsterdam, the Netherlands*

Microbiota study and fMRI study

Fredrik Bäckhed, MD, PhD – researcher, *University of Gothenburg, Gothenburg, Sweden and University of Copenhagen, Copenhagen, Denmark*

Kristien Fluitman, MD – researcher, *Amsterdam UMC, location VUmc, Amsterdam, the Netherlands*

Richard Ijzerman, MD, PhD – researcher, *Amsterdam UMC, location VUmc, Amsterdam, the Netherlands*

Bart Keijser, PhD – researcher, *TNO earth, Zeist, the Netherlands and Academic Center for Dentistry Amsterdam, the Netherlands*

Max Nieuwdorp, MD, PhD – principal investigator of the microbiota study and fMRI study, *Amsterdam UMC, location AMC and location VUmc, Amsterdam, the Netherlands*
