# Supplementary material for: Effectiveness and cost-effectiveness of personalised dietary advice aiming at increasing protein intake on physical functioning in community-dwelling older adults with lower habitual protein intake: rationale and design of the PROMISS randomised controlled trial
Source: BMJ Open. 2020 Nov 20;10(11):e040637. doi: 10.1136/bmjopen-2020-040637 (PMC7682452; doi:10.1136/bmjopen-2020-040637)
Supplement: Supplementary data [file bmjopen-2020-040637supp002.pdf]

BMJ Publishing Group Limited (BMJ) disclaims all liability and responsibility arising from any reliance placed on this supplemental material which has been supplied by the author(s)

Toestemmingsverklaring hoofdstudie

Toestemmingsformulier proefpersoon

Effectiviteit van het verhogen van eiwitinname op fysiek functioneren

- Ik heb de informatiebrief gelezen. Ook kon ik vragen stellen. Mijn vragen zijn voldoende beantwoord. Ik had genoeg tijd om te beslissen of ik meedoe.
- Ik weet dat meedoen vrijwillig is. Ook weet ik dat ik op ieder moment kan beslissen om toch niet mee te doen of te stoppen met het onderzoek. Daarvoor hoef ik geen reden te geven.
- Ik geef toestemming voor het informeren van mijn huisarts dat ik meedoe aan dit onderzoek.
- Ik geef toestemming voor het informeren van mijn huisarts over eventuele afwijkingen die tijdens het onderzoek gevonden worden.
- Ik geef toestemming voor het opvragen van informatie bij mijn huisarts over mijn nierfunctie, mochten er twijfels over de effecten op mijn gezondheid bestaan.
- Ik weet dat sommige mensen mijn gegevens kunnen inzien. Die mensen staan vermeld in deze informatiebrief.
- Ik geef toestemming voor het verzamelen en gebruiken van mijn gegevens op de manier en voor de doelen die in de informatiebrief staan.
- Ik geef toestemming om mijn gegevens op de onderzoekslocatie nog 15 jaar na dit onderzoek te bewaren.
- Ik wil meedoen aan dit onderzoek.

Naam proefpersoon:

Handtekening: Datum : __ / __ / __

Ik verklaar dat ik deze proefpersoon volledig heb geïnformeerd over het genoemde onderzoek.

Als er tijdens het onderzoek informatie bekend wordt die de toestemming van de proefpersoon zou kunnen beïnvloeden, dan breng ik hem/haar daarvan tijdig op de hoogte.

Naam onderzoeker (of diens vertegenwoordiger):

Handtekening: Datum: __ / __ / __

Reinders I, *et al. BMJ Open* 2020; 10:e040637. doi: 10.1136/bmjopen-2020-040637
