# Supplementary material for: Effectiveness and cost-effectiveness of personalised dietary advice aiming at increasing protein intake on physical functioning in community-dwelling older adults with lower habitual protein intake: rationale and design of the PROMISS randomised controlled trial
Source: BMJ Open. 2020 Nov 20;10(11):e040637. doi: 10.1136/bmjopen-2020-040637 (PMC7682452; doi:10.1136/bmjopen-2020-040637)
Supplement: Author's manuscript [file bmjopen-2020-040637.draft_revisions.pdf]

BMJ Open

BMJ Open is committed to open peer review. As part of this commitment we make the peer review history of every article we publish publicly available.

When an article is published we post the peer reviewers' comments and the authors' responses online. We also post the versions of the paper that were used during peer review. These are the versions that the peer review comments apply to.

The versions of the paper that follow are the versions that were submitted during the peer review process. They are not the versions of record or the final published versions. They should not be cited or distributed as the published version of this manuscript.

BMJ Open is an open access journal and the full, final, typeset and author-corrected version of record of the manuscript is available on our site with no access controls, subscription charges or pay-per-view fees (http://bmjopen.bmj.com).

If you have any questions on BMJ Open's open peer review process please email info.bmjopen@bmj.com

BMJ Open

Effectiveness and cost-effectiveness of personalized dietary advice aiming at increasing protein intake on physical functioning in community-dwelling older adults with lower habitual protein intake: rationale and design of the PROMISS trial

Journal:	*BMJ Open*
Manuscript ID	bmjopen-2020-040637
Article Type:	Protocol
Date Submitted by the Author:	18-May-2020

Complete List of Authors:	Reinders, Ilse; Vrije Universiteit Amsterdam,<br>Wijnhoven, Hanneke; Vrije Universiteit Amsterdam<br>Jyvakorpi, S; University of Helsinki<br>Suominen, Merja; University of Helsinki<br>Niskanen, Riikka; University of Helsinki<br>Bosmans, J; Vrije Universiteit Amsterdam<br>Brouwer, Ingeborg; Vrije Universiteit Amsterdam<br>Fluitman, Kristien; Amsterdam UMC; University of Gothenburg<br>Klein, Michel; Vrije Universiteit Amsterdam<br>Kuijper, Lothar; Vrije Universiteit Amsterdam<br>van der Lubbe, Laura; Vrije Universiteit Amsterdam<br>Olthof, Margreet R.; Vrije Universiteit Amsterdam<br>Pitkala, Kaisu H.; University of Helsinki<br>van der Pols-Vijlbrief, Rachel ; Vrije Universiteit Amsterdam<br>Visser, Marjolein; Vrije Universiteit Amsterdam

Keywords:	GERIATRIC MEDICINE, NUTRITION & DIETETICS, PUBLIC HEALTH

SCHOLARONE™
Manuscripts

I, the Submitting Author has the right to grant and does grant on behalf of all authors of the Work (as defined in the below author licence), an exclusive licence and/or a non-exclusive licence for contributions from authors who are: i) UK Crown employees; ii) where BMJ has agreed a CC-BY licence shall apply, and/or iii) in accordance with the terms applicable for US Federal Government officers or employees acting as part of their official duties; on a worldwide, perpetual, irrevocable, royalty-free basis to BMJ Publishing Group Ltd ("BMJ") its licensees and where the relevant Journal is co-owned by BMJ to the co-owners of the Journal, to publish the Work in this journal and any other BMJ products and to exploit all rights, as set out in our licence.

The Submitting Author accepts and understands that any supply made under these terms is made by BMJ to the Submitting Author unless you are acting as an employee on behalf of your employer or a postgraduate student of an affiliated institution which is paying any applicable article publishing charge ("APC") for Open Access articles. Where the Submitting Author wishes to make the Work available on an Open Access basis (and intends to pay the relevant APC), the terms of reuse of such Open Access shall be governed by a Creative Commons licence – details of these licences and which Creative Commons licence will apply to this Work are set out in our licence referred to above.

Other than as permitted in any relevant BMJ Author's Self Archiving Policies, I confirm this Work has not been accepted for publication elsewhere, is not being considered for publication elsewhere and does not duplicate material already published. I confirm all authors consent to publication of this Work and authorise the granting of this licence.

For peer review only

Effectiveness and cost-effectiveness of personalized dietary advice aiming at increasing protein intake on physical functioning in community-dwelling older adults with lower habitual protein intake: rationale and design of the PROMISS trial

Ilse Reinders[1], Hanneke A.H. Wijnhoven[1], Satu K. Jyväkorpi[2], Merja H. Suominen[2], Riikka Niskanen[2], Judith E. Bosmans[1], Ingeborg A. Brouwer[1], Kristien S. Fluitman[3,4], Michel C.A. Klein[5], Lothar D. Kuijper[1], Laura M. van der Lubbe[5], Margreet R. Olthof[1], Kaisu H. Pitkälä[2], Rachel Vijlbrief[1] and Marjolein Visser[1].

[1]Department of Health Sciences, Faculty of Science, and the Amsterdam Public Health research institute, Vrije Universiteit Amsterdam, The Netherlands; [2] University of Helsinki, Department of General Practice and Primary Health Care, and Helsinki University Central Hospital, Unit of Primary Health Care, Finland; [3]Department of Internal Medicine, Amsterdam UMC, Vrije Universiteit Amsterdam, Amsterdam Public Health research institute, Amsterdam, The Netherlands; [4]Wallenburg Laboratory, Department of Molecular and Clinical Medicine, Sahlgrenska Academy, University of Gothenburg, Gothenburg, Sweden; [5]Department of Computer Science, Vrije Universiteit Amsterdam, Amsterdam, the Netherlands.

Corresponding author:
Ilse Reinders
Vrije Universiteit Amsterdam
De Boelelaan 1105, kamer O-526
1081 HV Amsterdam
The Netherlands

For peer review only

ilse.reinders@vu.nl

Tel: +31205982467

Total word count: 7261

For peer review only

Abstract

Background: Short-term metabolic and observational studies suggest that protein intake above the recommended dietary allowance of 0.83 g/kg body weight (BW)/d may support preservation of lean body mass and physical function in old age, but evidence from RCTs is inconclusive.

Methods: The PROMISS trial examines effects of personalized dietary advice aiming at increasing protein intake with or without advice regarding timing of protein intake to close proximity of any usual physical activity, on change in physical functioning after 6 months among community-dwelling older adults (≥ 65y) with a habitual protein intake of < 1.0 g/kg adjusted (a)BW/d. Participants (n=264) will be recruited in Finland and the Netherlands, and will be randomized into three groups; two intervention groups and one control group. Intervention group 1 (n=88) receives personalized dietary advice and protein enriched food products in order to increase their protein intake to at least 1.2 g/kg aBW/d. Intervention group 2 (n=88) receives the same advice as intervention group 1, and advice to consume 7.5-10 g protein through protein (en)rich(ed) foods within half an hour after performing any usual physical activity. The control group (n=88) receives no intervention. All participants will be invited to attend lectures not related to health. The primary outcome is 6-months change in physical functioning measured by change in walk time using a 400-m walk test. Secondary outcomes are: 6-months change in the Short Physical Performance Battery score, muscle strength, body composition, self-reported mobility limitations, quality of life, and incidence of frailty, sarcopenia and malnutrition. We also investigate cost-effectiveness by change in health care costs.

Discussion: The PROMISS trial will provide evidence whether increasing protein intake, and additionally optimizing timing of protein intake, has a positive effect on the course of

For peer review only

physical functioning among community-dwelling older adults with a habitual protein intake of < 1.0 g/kg aBW/d.

Strengths and limitations of this study

- The effectiveness of advising to increase protein intake on 6-month change in physical functioning will be investigated;
- the combined benefit of increasing protein intake and timing of protein intake will be investigated;
- Only community-dwelling older adults with a habitual protein intake of < 1.0 g/kg aBW/d will be included;
- The intervention is based on personalized dietary advice;
- The biological value of the total protein intake will not be known.

Trial registration: ClinicalTrials.gov. Number of identification: NCT03712306. Registered 19 October 2018, https://clinicaltrials.gov/ct2/show/NCT03712306?cond=protein&cntry=NL&city=Amsterdam&draw=2&rank=1

Keywords: older adults, protein intake, physical functioning, RCT, malnutrition, protein recommendation, 400 meter walk.

For peer review only

Background

There is an ongoing debate on whether or not older adults should be recommended a protein intake above the current recommended daily allowance (RDA) established by the European Food Safety Authority (EFSA) of 0.83 g/kg body weight (BW)/d for adults (1). International panels of geriatricians, nutritional experts and scientists have proposed at least 1.0-1.2 g protein/kg BW/d for healthy older adults in order to maintain and regain muscle mass, strength and function (2, 3).

The proposed increase of the RDA for older adults is merely based on results from short term metabolic and epidemiological studies. Several metabolic studies showed that older adults (≥ 65 y) have a lower muscle protein synthesis (MPS) following protein intake compared to younger adults (4-6), and that higher protein intake enhances MPS in older adults when compared to lower protein intake (1.2 g/kg BW/d vs. 0.8 g/kg BW/d (7), or ≥ 30 g/d vs. 15 g/d (8)). In addition, the anabolic threshold (i.e. optimal dose of dietary protein in a meal that stimulates MPS) is 70% higher in older compared to younger adults (5). Epidemiological studies have shown that higher dietary protein intake in older adults, defined as > 0.9 g/kg BW/d (9) or > 1.0 g/kg BW/d (10-12) is associated with lower risk of weight loss (11), better disability trajectories (12), less loss of lean mass (9), or lower risk of developing functional impairments (10).

Despite the evidence from metabolic and epidemiological studies, causal evidence to support beneficial effects of protein intake at or above 1.0 g/kg BW/d based on randomized controlled trials (RCTs) is not conclusive. One systematic review showed no beneficial effect of increasing protein intake on lean body mass, muscle cross-sectional area, muscle strength, or physical performance (13). Of the 36 studies included in the systematic review, 26 studies presented mean habitual protein intake of the study participants which ranged between 0.78 and 1.5 g/kg BW/d, with only one study below the protein RDA of 0.8 g/kg BW/d (13). The

For peer review only

relatively high mean habitual protein intake may explain the absence of a beneficial effect of additional protein. Another explanation may be that the amount of protein provided might not have been sufficient to augment MPS. I.e. a protein intake of 25 – 30 g is required to stimulate MPS and maintain muscle mass (14, 15), though the amounts provided varied between 10 g/d (3d/wk) and total intake of 125 g/day or were not reported. Of the trials published after the systematic review, Park et al. showed that intake of 1.5 g/kg BW/d for 12 weeks resulted in higher muscle mass and improved gait speed compared to intake of 0.8 g/kg BW/d in undernourished pre-frail and frail older adults (16). Ten Haaf et al. showed a positive effect of increasing protein intake for 12 weeks on lean body mass in active older adults with a habitual protein intake of < 1.0 g/kg BW/d (17). Beelen et al. found no effects of protein supplementation on physical performance among older adults after hospital discharge (18), however, baseline protein intake was already 1.0 g/kg BW/d in the control group and 1.5 g/kg BW/d in the intervention group. Finally, Bhasin et al. showed no beneficial effects other than a decrease in fat mass after a controlled diet with 1.3 g/kg BW/d of protein for 6 months compared to a control diet consisting of 0.8 g protein /kg BW/d (19) among functionally limited community-dwelling men aged ≥ 65 years. However, mean BMI of the participants was quite high (30.3 kg/m^2), which may have resulted in an overestimation of baseline protein requirements. Based on inconsistent findings, more RCTs in older adults with lower habitual protein intake are needed to determine the potential effect of increasing protein intake on physical functioning outcomes.

Previous studies among older adults showed that protein supplementation in combination with resistance exercise has more beneficial effects on body composition, muscle strength and physical function compared to resistance exercise alone (20-26). The underlying hypothesis is that protein supplementation augments the adaptive response of skeletal muscle to resistance exercise. In addition, there is evidence that timing of protein

For peer review only

For peer review only - http://bmjopen.bmj.com/site/about/guidelines.xhtml

intake in close proximity of physical activity stimulated MPS to greater extent than when timed at other hours during the day (27). To our knowledge, there are no RCTs investigating the effect of timing protein intake in in close proximity of physical activities on physical functioning.

The Prevention Of Malnutrition In Senior Subjects in the EU (PROMISS) trial is designed to fill in some of the current knowledge gaps on the optimal amount of dietary protein in older community dwelling adults and timing of protein intake in relation to physical activity. Its primary objective is to examine the effectiveness of personalized dietary advice aiming at increasing protein intake to at least 1.2 g/kg adjusted (a)BW/d on change in physical functioning after 6 months measured by change in walk time using a 400-m walk test among community-dwelling older adults with a habitual protein intake of < 1.0 g/kg aBW/d. Additionally, it examines the combined effect of personalized dietary advice aiming at increasing protein intake to at least 1.2 g/kg aBW/d and advice aiming at optimizing the timing of protein intake in close proximity of any usual physical activity. The secondary objectives are to examine the effectiveness of personalized dietary advice aiming at increasing protein intake to at least 1.2 g/kg adjusted on 6-month changes in physical functioning measured by the Short Physical Performance Battery (SPPB) score, muscle strength, body composition, self-reported mobility limitations, quality of life, incidence of frailty, incidence of sarcopenia, and incidence of malnutrition and change in health care costs.

In three ancillary studies the following additional objectives are addressed; 1) the effect of using persuasive technology on adherence to personalized dietary advice aiming at increasing protein to at least 1.2 g/kg aBW/d, 2) the effect of personalized dietary advice aiming at increasing protein intake to at least 1.2 g/kg aBW/d on the oral and gut microbiota composition, and 3) the effect of personalized dietary advice aiming at increasing protein

For peer review only

intake to at least 1.2 g/kg aBW/d on central neural responses to food-cues in brain areas of interest.

Methods/Design

Study design

The PROMISS trial is a multicentre randomized controlled trial (ClinicalTrials.gov identifier: NCT03712306) designed to examine the effectiveness of personalized dietary advice aiming at increasing protein intake and advice on optimizing the timing of protein intake in close proximity of any usual physical activity on change in physical functioning after 6 months. Participants will be randomised into three groups: one control group (no intervention); intervention group 1) receiving personalized dietary advice aiming at increasing protein intake to at least 1.2 g/kg aBW/d; and intervention group 2) receiving personalized dietary advice aiming at increasing protein intake to at least 1.2 g/kg aBW/d, including personalized advice to optimize the consumption of protein in close proximity of any usual physical activity.

Researchers, nutritionists, and statisticians are responsible to the design of the trial. Older adults were not involved in the design or conduct of the research.

Eligibility criteria

The eligibility criteria are proposed to include a study group of older adults (65+) with a habitual protein intake < 1.0 g/kg aBW/d. Inclusion and exclusion criteria are listed in Table 1, and some are described in more detail below.

Older adults with a BMI of < 18.5 kg/m^2 will be excluded, because these participants are likely to be undernourished (28) and should preferably receive general nutritional care that is not provided in this trial. Those with a BMI of > 32.0 kg/m^2 will be also excluded,

For peer review only - http://bmjopen.bmj.com/site/about/guidelines.xhtml

173 because they should be advised to lose weight, which is not the aim of the present study and
174 may interfere with the study objective. Because participants of intervention group 2 will be
175 advised to consume protein rich foods in close proximity of any usual physical activity, older
176 adults who are bedridden, wheelchair bound or do not go outside will be excluded from the
177 trial. Older adults with a diagnosis of severe kidney disease (i.e. treatment of a nephrologist
178 and/or protein-restricted diet, self-reported) will also be excluded as they should be advised to
179 limit their protein intake (2, 29-31). Older adults with a low cognitive status (Mini-Mental
180 State Examination (MMSE) score ≤ 20 (32)) will be excluded, as participants should be able
181 to understand and follow dietary advice if randomized to one of the intervention groups.
182
183 Calculation of protein intake using adjusted body weight
184 To calculate habitual protein intake in g/kg aBW/d for all (potential) participants and
185 recommended protein intake (participants in the two intervention groups), we apply adjusted
186 BW depending on participants' age and BMI. We use adjusted body weight because
187 underweight persons require extra protein to build muscle tissue, while in overweight
188 persons, much 'extra weight' is adipose tissue. Protein intake in g/kg aBW/d is based on self-
189 reported BW during screening and afterwards based on measured BW during the baseline
190 assessment, which is further used throughout the study. For those with a BMI > 25.0 to 32.0
191 kg/m^2 (age ≤ 70 y) or > 27.0 to 32.0 kg/m^2 (age > 70 y) we apply aBW corresponding to a
192 BMI of respectively 25.0 or 27.0 kg/m^2. For those with a BMI > 18.5 to < 22.0 kg/m^2 (age >
193 70 y) we apply aBW corresponding to a BMI of respectively 18.5 or 22.0 kg/m^2 (33). For the
194 recommended protein intake, we apply adjusted BW which is based on baseline measured
195 BW.
196
197 *Recruitment and screening*

For peer review only - http://bmjopen.bmj.com/site/about/guidelines.xhtml

Two hundred and sixty-four community-dwelling adults aged 65 years and older will be recruited at two study sites (metropolitan area of Finland including Helsinki, Espoo, Vantaa, Kauniainen, and Amsterdam, the Netherlands). The recruitment strategy includes mass mailing using addresses obtained from a random sample of the Finnish Population Registry (in Finland only), newspaper advertisements, media coverage, lectures, oral presentations to the target group, informing professionals working with older adults and flyers which will be distributed at locations where many community-dwelling older adults visit.

Older adults who are interested in participating will be asked to contact the local PROMISS research team (by phone or by e-mail). Thereafter, screening by phone takes place, only when verbal informed consent is given, in which the majority of the eligibility criteria will be assessed along with an explanation of the study. Only those with a lower habitual protein intake (< 1.0 g/kg aBW/d) will be invited for the first clinic visit. Assessment of habitual protein intake will be estimated in two steps: 1) initial screening by phone; 2) a full dietary assessment based on a combination of three food diaries and three 24-h dietary recalls to confirm lower habitual protein intake. Step 1, the initial screening is performed by phone using the Protein Screener 55+ (Pro55+, available for use in English, Finnish and Dutch: see www.proteinscreener.nl/#/). This screening tool was specifically developed and validated for this purpose (34). The screening results in a probability score (0-100%) of having a protein intake below 1.0 g/kg aBW/d. At a probability of > 30%, sensitivity and specificity are optimally balanced (34). In the PROMISS trial we select persons with a probability score varying between > 15% (when initial response rates to recruitment strategies are low) and > 30% (when initial response rates are high), for the second step of assessing habitual protein intake. Those who fulfill the eligibility criteria receive further information on the study and a food diary with a booklet with pictures of portion sizes by post to support the 24-h dietary recalls. After a minimum of one week of consideration, the

For peer review only

For peer review only - http://bmjopen.bmj.com/site/about/guidelines.xhtml

research staff contacts the older adults, and among those who are still willing to participate the full dietary assessment will take place (step 2). These potential participants will be asked to keep track of their dietary intake by filling out the provided food diary for three consecutive days (three weekdays; or two weekdays and one weekend day). The booklet with pictures of portion sizes that they received earlier will help them accurately filling out the diary. Each day after, they will be called by a nutritionist to go through their food diary of the day before (24-h dietary recall). Potential participants are asked whether these days are representative for their habitual diet. In case one of the three days is not representative, mean protein intake is based on two instead of three days. In case of more than one non-representative day, the person will be excluded. The food intake data based on the 24-h dietary recall will be entered into the program 'Fineli' for the Finnish data (35) and into the program 'Eetmeter' of the Dutch Nutrition Center using an extended version of the Dutch Food Composition Table of 2016 for the Dutch data (36) to calculate intake of macronutrients and micronutrients (vitamin D and vitamin B12). Participants with an actual protein intake ≥ 1.0 g/kg aBW/d (based on self-reported BW) will be excluded.

Potential participants with a mean habitual protein intake < 1.0 g/kg aBW/d (based on the three 24-h dietary recalls), will be invited for the clinic visit, where final eligibility criteria will be assessed; MMSE > 20, ability to walk 400 m within 15 minutes (the use of a cane is allowed, but without the use of a walker and no rest longer than 60 seconds), and BMI of ≥ 18.5 kg/m^2 and ≤ 32.0 kg/m^2 based on measured BW and body height. When all eligibility criteria are met, participants are included in the PROMISS trial.

Randomization, allocation and masking

Randomization by means of a stratified block randomization procedure will be performed by an independent statistician. Participants will be allocated in a 1:1:1 ratio to the three groups.

For peer review only - http://bmjopen.bmj.com/site/about/guidelines.xhtml

The size of the randomization blocks is three. Participants will be stratified according to their baseline habitual protein intake (< 0.9 or 0.9-1.0 g/kg aBW/d) and sex to ensure homogeneous distribution of baseline habitual protein intake and sex in the three groups across the two recruitment sites, because there may be a different intervention effect by baseline habitual protein or sex. In case couples are eligible we will allocate them to the same intervention group to limit interference between intervention groups. We will randomly select on which partner the randomization for the intervention group is based. Any resulting unbalance in the number of subjects per treatment arm will be corrected in the randomization of the next block. Due to the nature of the study, researchers, nutritionists and participants are not blinded to the study group.

Study timeline

The first clinic visit starts with written informed consent, and when participants are eligible, the baseline assessment will be performed. The baseline assessment consists of questionnaires (frailty status, risk of sarcopenia, self-reported mobility limitations, quality of life (QoL) and health care costs) and measurements (physical function, muscle strength and body composition). See below 'primary and secondary outcomes' and 'other measures' for details on these assessments. An accelerometer will be attached to measure physical activity for 7 subsequent days.

After the baseline assessment, participants will be randomised to one of the three study groups done by the nutritionists and they will inform the participants in which group they are allocated to. Participants randomized to one of the two intervention groups will be invited for a consultation meeting at the clinic to receive their personalized dietary advice, and personalized advice on optimizing the timing of protein intake in close proximity of any usual physical activity (intervention group 2 only). This will take place within 2 weeks after

For peer review only

For peer review only - http://bmjopen.bmj.com/site/about/guidelines.xhtml

the baseline assessment since the personalized advice needs to be composed by the nutritionist. The baseline assessment is considered the start of the study period for participants of the control group, while the consultation meeting is considered the start of the study period for participants of the intervention groups.

One week prior to the 3-month follow-up visit, dietary intake will be assessed again by means of a combination of three food diaries and three 24-h dietary recalls. The 3-month follow-up visit will take place at the clinic and includes measurement of BW, assessment of self-reported mobility limitations, risk of sarcopenia, QoL, health care costs and the accelerometer will be attached to measure physical activity for 7 subsequent days.

One week prior to the 6-month follow-up visit (final measurement) dietary intake will again be assessed by means of a combination of three food diaries and three 24-h dietary recalls, which allows us to determine compliance to the dietary advice. The 6-month follow-up visit at the clinic includes all measurements performed during the baseline visit and the accelerometer is attached again to measure physical activity for the next 7 days. Finally, among participants of the intervention groups only, several questions regarding the appreciation and adherence of the intervention and participants' intention to follow the dietary advice in the future will be asked in order to perform a process-evaluation. Figure 1 shows the study timeline and Table 2 provides an overview of all measurements.

Intervention

Participants in the intervention group 1 will receive a personalized dietary advice by nutritionists dedicated to this study aiming at increasing their protein intake to at least 1.2 g/kg aBW/d (without increasing total daily energy intake), based on their habitual dietary characteristics, protein intake and measured BW assessed at baseline. The advice includes the use of regular protein rich food products and protein enriched food products provided by the

For peer review only - http://bmjopen.bmj.com/site/about/guidelines.xhtml

research team, and will be based on personal dietary preferences. Protein enriched food products that can be incorporated within the regular diet include bars, cereals, puddings, coconut water and whey powder, which will be freely provided and shipped to participants' home. Those products can be incorporated in the dietary advice as they can make it easier to increase protein intake due to their high protein content. Participants receive guidelines how to incorporate the protein enriched food products within their diet. The dietary advice will also incorporate the advice to consume at least one daily meal consisting of ≥ 35 g protein, as studies have shown that this amount increases MPS in older adults (37-39).

Participants in intervention group 2 will receive the same dietary advice as intervention group 1, and the personalized advice to consume at least 7.5-10 g protein through protein (en)rich(ed) food products within half an hour after performing any usual physical activity as this may enhance resistance exercise induced MPS (27). One RCT among older adults has shown that protein supplementation in combination with resistance exercise had beneficial effects on e.g. muscle mass and function, but no differences in effect were found between protein consumption pre versus post resistance exercise (40). We therefore recommend protein intake after physical activity as this is a uniform and more feasible advice compared to 'in close proximity of', and might also result in less stomach discomfort as compared to protein consumption prior to physical activity. Usual physical activity is defined as either physical exercise (e.g. biking, swimming, tennis) or the most intensive activities of daily living when the participant does not engage in physical exercise (e.g. gardening, housekeeping, doing groceries) for a minimum of 30 minutes. The advice is linked to most extensive or longest physical activity. Participants are instructed not to become more or less physically active but merely to shift their physical activity or protein intake moment.

During the intervention period, nutritionists will plan follow-up phone calls in consultation with the participants during week 2, week 4, week 8, week 16 and week 20 to

For peer review only

ask if they have understood the advice and are able to adhere to the advice. In addition, any issues related to the use of protein enriched food product can be discussed (intervention groups only). If necessary, changes in the dietary advice will be made, for example when weight change > 2 kg has occurred (based on self-assessment). Participants allocated to the control group do not receive any intervention, but are contacted on similar time points as the intervention groups to ask how they are doing.

All participants are invited to a minimum of one organized lecture on non-health related themes and other social events during the trial in order to stimulate their commitment to the trial. Separate lectures/events (with the same topic) are organized for the intervention groups and the control group to prevent interference between intervention arms.

Compliance

We will collect dietary intake prior to the 3-month follow-up visit (by means of the combination of three food diaries and three 24-h dietary recalls) to assess compliance to the dietary advice. This information allows the nutritionists to provide additional advice – if needed – for participants in the intervention groups, which will be provided during the 3-month follow-up visit. Dietary intake will again be assessed at follow-up and compliance to the dietary advice will be determined.

Intervention fidelity

To ensure good adherence to the intervention protocols at both study sites, all personnel working for the trial have undergone extensive training. The nutritionists will follow written standardized operational procedures to develop and provide the personalized dietary advice (with or without additional advice to consume protein within half an hour after any usual physical activity). Four times during the conduct of the trial, the nutritionists from one site

For peer review only - http://bmjopen.bmj.com/site/about/guidelines.xhtml

will visit the other site to attend assessments, and potentially notice and correct differences in order to ensure identical execution of the trial at both sites. In addition, monthly Skype-meetings will be held between all staff involved in the execution of the trial at both sites to solve any potential day-to-day issues in a standardized way. Furthermore, identical participant brochures and other printed materials have been developed and translated to Dutch and Finnish language.

Outcomes

<u>Primary outcome</u>

The primary outcome of the PROMISS trial is 6-months change in physical functioning measured by change in walk time using a 400-m walk test (Long Distance Corridor Walk) (41, 42). This test is predictive for higher risk of mortality, incident cardiovascular disease and mobility limitation and disability (43). One advantage of this continuous outcome is that it enables discrimination between categories of risk among participants (44), and it is less prone to a ceiling effect as compared to other functional outcome measures (e.g. SPPB) (45). The course for the 400-m test is 20-m long and marked by a traffic cone and tape line at the beginning and end. For all participants, the test will begin with a mandatory 40-m walk (warm-up) at their usual pace. Thereafter the 400-m test starts with the feet behind and just touching the starting line and ends after 10 complete rounds when one foot is behind the end line. For the 400-m test, older adults will be instructed to walk as fast as possible at a pace they can maintain for 400 m. Standardized encouragement will be given each lap, including the number of laps remaining. At the 6-month follow-up visit, older adults are allowed to use a cane, can take rest as needed (but no rest longer than 60 seconds) and there will be a time limit of 17 minutes. Time will be recorded to the nearest second.

For peer review only

Secondary outcomes

The secondary outcomes are changes in physical functioning, muscle strength, body composition, self-reported mobility limitations, QoL, incidence of frailty, incidence of sarcopenia, and incidence of malnutrition. We will also investigate change in health care costs.

Physical functioning will be assessed by means of the Short Physical Performance Battery (SPPB) (46). The SPPB assesses lower extremity function which consists of three timed tests: repeated (5x) chair stands test, 4-meter walk test and three standing balance tests (ability to stand with the feet together in the side-by-side, semi-tandem, and tandem positions). The total score ranges from 0-12. A higher score indicates better physical functioning.

Muscle strength will be determined by hand-grip strength (kg). Hand-grip strength is an indicator of overall muscle strength (47) and a higher hand grip strength is associated with decreased risk of physical disabilities (48) and all-cause mortality in old age (48, 49). Maximum grip strength will be measured three times at each hand during baseline and 6-month follow-up visit. We will use a digital dynamometer (Saehan Digital Hand held dynamometer) adjusted for hand size. Participants will be measured in an upright sitting position with the forearms supported by the armrest of a chair according to a standardized protocol (50). The mean of the maximum score of left and right hand will be used for analyses. Muscle strength will also be determined by leg extension strength (N). A higher leg extension strength is associated with decreased risk of mobility disability (51, 52) and higher risk of early mortality (53-55). Leg extension strength will be assessed using a chair designed to measure leg extension strength (56). Maximum leg extension strength will be measured three times for each leg during baseline and 6-month follow-up visit. The mean of the maximum score of left and right leg will be used for analyses.

For peer review only - http://bmjopen.bmj.com/site/about/guidelines.xhtml

398 Body composition will be estimated by means of bioelectrical impedance using the BodyStat 1500MDD devise, using the Kyle equation to determine fat percentage (%), fat mass and fat-free mass (60) and the Sergi equation to determine appendicular skeletal muscle mass (ASMM, kg) (61). Additionally, at the Dutch study site, body composition (fat free mass (kg), fat mass and fat percentage (%)) will be measured by air displacement plethysmography (62).

404 Self-reported mobility limitations will be assessed by means of a questionnaire; "Because of your health, how much difficulty do you have walking 400 meter?" and "Because of your health, how much difficulty do you have climbing 10 steps?" Participants will respond using a five level Likert scale: 'no difficulty', 'a little difficulty', 'some difficulty', 'a lot of difficulty' and 'unable to do the activity'.

409 QoL will be measured using the EuroQol 5D – 5L questionnaire (63).

410 Incident frailty will be assessed using the Fried criteria (57). Participants will be considered 'frail' when three or more components are present. Those with no components will be considered 'robust', whereas those with one or two components will be considered 'prefrail'. The criteria include 1) self-reported unintentional weight loss (> 4 kg in past 6 months), 2) self-reported exhaustion (based on two questions from the Center for Epidemiologic Studies Depression (CES-D) scale on exhaustion in the past week at baseline and follow-up: "I felt that everything I did was an effort" and "I could not get going". Scores ranges from 1 'rarely or none of the time' to 4 'always or most of the time'. A score of 3 or 4 on either question indicates exhaustion (58), 3) weakness (grip strength in the lowest 20% of the whole study population based on the mean of the maximum scores, adjusted for gender and BMI), 4) slow walking speed (walk time on the 4m walk test in the slowest 20% of the whole study population, adjusted for gender and height), and 5) low physical activity (total counts per week based on the accelerometer data in the lowest 20% of physical activity for

For peer review only

For peer review only - http://bmjopen.bmj.com/site/about/guidelines.xhtml

each gender). Incident frailty is considered deterioration of frailty status; i.e. from robust at baseline to pre-frail or frail at follow-up or from pre-frail at baseline to frail at follow. Frail participants at baseline will be excluded from these analyses.

Incidence of sarcopenia will be assessed with the SARC-F questionnaire (59); how much effort do you experience when 1) lifting and carrying a bag of 4.5 kilo, 2) walking across a room, 3) transferring from a chair or bed, 4) climbing a flight of 10 stairs, and 5) how many times have you fallen in the past year. Answering option include no effort (0 points), a bit of effort (1 point) and a lot of effort (2 points), where a score equal to or greater than 4 is predictive of sarcopenia and poor outcomes. Participants with sarcopenia at baseline will be excluded from these analyses.

Incidence of malnutrition will be defined as BMI < 22.0 kg/m^2 or unintentional weight loss > 5% in the last 6 months. Malnourished participants at baseline will be excluded from these analyses.

A modified version of the Resource Utilization in Dementia Questionnaire (RUD) (64) will be used to collect data on health care and social utilization costs over the period three month prior to baseline, three months prior to the 3-month follow-up visit and three months prior to 6-month follow-up visit. Costs include costs of primary and secondary care, complementary care, informal care and home care.

Other measures

BW will be measured without shoes in underwear to the nearest 0.1 kg using a digital calibrated scale (Finland; SECA 877, the Netherlands; Marsden M-520). Body height will be measured to the nearest millimeter using a SECA stadiometer for mobile height measurements (Finland; SECA 217, the Netherlands; SECA 214). Corrections will be made to adjust the measured body weight for clothing, shoes or a cast (minus 1 kg for each

For peer review only - http://bmjopen.bmj.com/site/about/guidelines.xhtml

element), and to adjust the measured body height for shoes (minus 1 cm). Physical activity will be objectively assessed by means of an accelerometer (Axivity, AX3) during 7 subsequent days after each clinic visit (baseline, 3-month follow-up visit and 6-month follow-up visit). The accelerometer will be attached by a nutritionist to the frontal part of the right thigh in the mid-point between iliac crest and patella bone when sitting down, with a surgical plaster. Participants can perform any physical activity as the accelerometer is water resistant. Appetite will be measured with SNAQ-Appetite questionnaire (65). Dietary intake will be assessed by means of a combination of three food diaries and three 24-h dietary recalls prior the 3-month follow-up visit and the 6-month follow-up visit.

Sample size and statistical analyses

<u>Sample size</u>

The study is powered to detect a substantial meaningful change of 28 sec (SD=61 sec) (66) between the respective intervention groups and the control group on the primary outcome walk time on the 400-m walk test, assuming a 2-sided test at $\alpha=0.05$ with a power of 0.8. For this, 75 participants per group are needed, which is 225 in total. Assuming a drop-out of 15% (which was reported in a comparable study of Bhasin et al. 2018 (19), the total number of study participants to be included in the study will be n=264. Thus, a total of n=132 at each study site (n=44 per group per study site).

<u>Statistical analyses plan</u>

Statistical reporting will be according to the CONSORT standards (67). The collected data at the two study sites will be pooled together at the Amsterdam site, with a variable indicating study site. All statistical analyses on primary and secondary outcome measures will be

For peer review only

performed by an independent statistician blinded for group allocation. Baseline characteristics will be described (percentages, means ± standard deviations) by study group.

The primary analyses will be based on the intention-to-treat principle, i.e. data from participants allocated to the intervention groups will be analyzed as part of those groups, irrespectively of their level of adherence to the advice. Multiple Imputation using multivariate Imputation by Chained Equations will be used to impute missing cost and effect data. The continuous primary outcome (change in 400-m walk time) will be analyzed using mixed model regression analyses with study site and participating partner as random variable. We will adjust for baseline 400-m walk time as well as baseline protein intake (g/kg aBW/d) and sex (the stratification factors for randomization). We will compare intervention effects of the respective intervention groups versus the control group. Effects will also be expressed in Cohen's d and the corresponding 95% confidence intervals will be calculated, which allows comparison between intervention effect estimates between different outcome measures. The secondary outcomes and other measures will be analyzed using mixed model regression analogously to the primary outcome. For binary secondary outcomes, generalized estimating equation models will be used. With regard to time-to-event analyses (incident sarcopenia and mobility limitations) Cox proportional hazard models will be used. Time-to-event is defined as the time of the start of the study period to the date of the first occurrence of the event (3-month follow-up visit or 6-month follow-up visit). Participants who do not meet these criteria will be censored at the latest time we had information available. We will perform subgroup analyses stratified by baseline protein intake (< 0.9 or 0.9-1.0 g/kg aBW/d), sex and baseline 400-m time (based on median) for the primary and secondary outcomes.

Per-protocol analyses will also be conducted as a sensitivity analysis. Effect estimates for change in primary and secondary outcome measures will be calculated for participants from the intervention groups who reached the protein target of at least 1.2 g/kg aBW/d after

For peer review only - http://bmjopen.bmj.com/site/about/guidelines.xhtml

both 3 and after 6 months (mean protein intake based on the three 24-h dietary recalls) vs. participants from the control group. Data will be analyzed using SPSS (IBM SPSS Statistics. Armonk, NY). A two-sided *P*-value of 0.05 is considered statistical significant.

The cost-effectiveness analysis will be performed from a healthcare perspective. Total mean costs during the study will be related to physical functioning (the 400-m walk test) and change in Quality-Adjusted Life-Years based on the EuroQol 5D questionnaire. Mixed model regression analyses will be used to estimate differences in the primary outcome of the respective interventions groups versus the control group. Linear regression analyses will be used to estimate differences in QoL (expressed as Quality-Adjusted Life-Years) and healthcare costs. Incremental cost-effectiveness ratios will be calculated by dividing the difference in costs by the difference in effects. Statistical uncertainty will be estimated using bias-corrected accelerated bootstrapping (5000 replications) and will be presented using cost-effectiveness planes and cost-effectiveness acceptability curves.

Participants' safety

In case any (medical) questions arise during the screening or intervention period, participants can consult an independent medical doctor. All adverse events and serious adverse advents will be tracked by the nutritionists during the follow-up phone calls, 3-month follow-up visit and 6-month follow-up visit to assess their potential relationship to the intervention at both sites and will documented in the final report. Adverse events will be reported within 7 days (death or life threatening situations) or within 15 days (in case of other adverse events) of first knowledge to The Medical Ethical Committee of the Amsterdam UMC, location Vumc (required for the Dutch site only).

Data quality assurance and data management

For peer review only - http://bmjopen.bmj.com/site/about/guidelines.xhtml

Research data will be collected at each site and each visit (baseline, 3-month follow-up visit and 6-month follow-up visit) using a standardized protocol with the same order of assessments, and entered twice in separate electronic datasets. When discrepancies between the datasets are found, the original questionnaire will be consulted. Questionnaire items and measurements will include the corresponding variable names to minimize errors in data entering. Finally the two final electronic dataset (one from each site) containing all data will be pooled. A data catalogue and codebook will be developed.

Original questionnaires will be stored in a secure manner at each site in an area with limited access. All records that contain names (i.e. informed consent forms), will be stored separately from study records identified by code number. All databases will be secured with password-protected access systems.

Ancillary studies

Within the PROMISS trial, three ancillary studies will be conducted: 1) persuasive technology study, 2) microbiota study, and 3) fMRI study.

<u>1. Persuasive technology study</u>

The primary aim of this study is to examine the effect of persuasive technology on adherence to the personalized dietary advice aiming at increasing protein intake to at least 1.2 g/kg aBW/d in a sub-sample of Dutch participants from intervention group 1 (n=24) and intervention group 2 (n=24), i.e. the first 24 participants of intervention group 1 and the first 24 participants of intervention group 2 that consent to it (writting informed consent will be signed).

Participants will be provided with a food storage box that registers which provided protein enriched food products are taken out. The food box is used to store the protein

For peer review only - http://bmjopen.bmj.com/site/about/guidelines.xhtml

enriched food products provided by the research team. Participants will also receive a tablet that allows participants to register any consumed protein enriched food products and supports them in finding alternative food products that contain a comparable amount of protein. For this, the system uses the personalized dietary advice as provided by the nutritionist and data from the storage box. The tablet application aims to stimulate adherence to the dietary advice by providing tailored and personalized messages. In addition, personality characteristics and communication style preferences that are determined via a questionnaire completed at baseline are used to tailor the style and tone of these messages (68).

In addition to the personalized messages, half of the participants from intervention group 1 and 2 who participate in the persuasive technology sub-study will also receive a gamified version of the tablet application (n=12 + n=12). In this version, participants can earn game-points by registering their consumed protein (en)rich(ed) food products and by playing mini-games about the protein content of foods (i.e. guess the protein content, more-or-less protein). The distribution of receiving the gamified version vs. standard version is quasi-randomized, where we will balance the group size.

At the consultation meeting, participants receive their food storage box and tablet. Both are fully configured, i.e. they are loaded with their personal dietary advice. After the 6-month follow-up visit, participants will be asked to return the equipment and fill out questions on the feasibility and user experience of the provided persuasive technology.

The secondary objectives are 1) to investigate to what extent participants perceive messages of which the style and tone are adapted to their personal characteristics as personalized and adequate, and 2) to determine the effect of gamification on the effectiveness and feasibility of the persuasive technology.

2. Microbiota study

For peer review only

In the microbiota study, the effect of personalized dietary advice aiming at increasing protein intake in community-dwelling older adults with lower habitual protein intake on both the oral and gut microbiota is investigated. The study will be conducted at both study sites.

The human microbiota consists of the $4*10^{13}$ micro-organisms that inhabit the body (69). The emergence of next generation DNA sequencing techniques at the start of the 21st century has allowed more detailed study of the microbiota and since then, the microbiota composition has been associated with both health and disease (70), as well as aging itself (71, 72). Moreover, several interventional studies proved that dietary changes also affect the gut microbiota, with the first microbial shifts being evident within 48 hours (73). The altered microbiota in turn, can differentially affect the human host metabolism through the production of metabolically active metabolites. Less is known about the oral microbiota. It was found to be associated with oral health and function and even nutritional status (74, 75), but its possible role in undernutrition in older adults has not been investigated.

A fresh frozen fecal sample and tongue swab is collected at baseline and 6-month follow-up visit once written informed consent is provided. Participants from either the control group or intervention group 1 can be included in this study. Participants from the intervention group 2 are excluded to limit the number of groups and parameters in this exploratory study. Additional exclusion criteria are: use of systemic antibiotics in the three months prior to the first sampling visit, diagnosis with inflammatory bowel disease and prolonged institutionalization (> 4 weeks) in the three months prior to the first sampling visit. There is no restriction other than consent rate to the number of PROMISS participants that will be included in this side study.

Once all samples from all participants are collected, fecal samples are shipped to the Wallenberg Laboratory of Cardiovascular and Metabolic research (at the University of Gothenburg, in Sweden) for 16S rRNA sequencing using sequencing methods previously

For peer review only - http://bmjopen.bmj.com/site/about/guidelines.xhtml

described (76). The tongue swabs will be send to the Netherlands Organisation for Applied Scientific Research for 16S rRNA sequencing as is previously described (77).

3. fMRI study

In the fMRI study, we will investigate the effect of personalized dietary advice aiming at increasing protein intake in community-dwelling older adults with lower habitual protein intake on central brain circuits involved in the regulation of appetite. Several studies demonstrated that increasing protein intake affects appetite (78) and the gut microbiota (79). However, none have studied the effects on both simultaneously, or the interaction. A functional MRI (fMRI) scan will be used to measure the brain responses to visual or actual food cues. Brain activity in response to food cues will also be related to (shifts) in the gut microbiota. Therefore, only participants from the microbiota side study can be included in this study, with additional exclusion criteria: being claustrophobic, being diagnosed with a mental disorder (e.g. depression or addiction), being uncorrectable visually or hearing impaired, or having a contra-indication for MRI-scans (e.g. having a pacemaker). Up to 50 participants will be included in this side study. This side study will only be conducted at the Dutch study site.

Once written informed consent is provided, participants who are included in this side study will be asked to visit the Amsterdam University Medical Centre, location VUmc, for an fMRI scan twice during the study period: at baseline and at 6-month follow-up visit. Prior to the fMRI-scan, additional salivary and blood samples will be collected for determination of additional nutritional and microbial biomarkers. The protocol for the fMRI experiments have been previously described (80, 81).

Discussion

For peer review only - http://bmjopen.bmj.com/site/about/guidelines.xhtml

There is an ongoing discussion whether the EFSA RDA of 0.8 g protein /kg BW/d is sufficient for older adults and whether it should be increased to at least 1.0-1.2 g protein/kg BW/d to support muscle health and functioning. National guidelines of some European countries already increased their RDA, i.e. the RDA of the German-speaking countries (D-A-CH) is increased to 1.0 g/kg BW/d (82), and the Nordic Nutrition Recommendation has increased their RDA to 1.2 g/kg BW/d (83). The PROMISS trial is the first RCT which will investigate the effect of personalized dietary advice aiming at increasing protein intake and the combined effect of personalized dietary advice aiming at increasing protein and optimally timing protein intake in close proximity of any usual physical activity, on change in physical functioning after 6 months among community-dwelling older adults (≥ 65 y) with a habitual protein intake of < 1.0 g/kg adjusted (a)BW/d. The PROMISS trial will therefore provide additional insight to the question whether the current EFSA RDA for protein for older adults should be increased to 1.2 g/kg aBW/d, and whether optimal timing of protein intake will additionally benefit physical functioning.

A strong and unique aspect of the PROMISS trial is that we will include participants with a habitual protein intake < 1.0 g/kg aBW/d, excluding those with a BMI < 18.5 and > 32.0 kg/m^2. This will allow us to examine the effects of increasing protein intake from < 1.0 to at least 1.2 g/kg aBW/d. An innovative component of our study is that we will investigate the combined benefit of increasing protein intake and timing of protein intake with any usual physical activity on physical functioning and other health related outcomes. Another strength is that in our study the intervention is based on personalized dietary advice which is likely more feasible in the long term to maintain in everyday life, compared to providing custom-prepared meals (19) or protein supplements (84, 85), as done in most other studies. Finally, we will be able to investigate the effect of persuasive technology on adherence to the dietary

For peer review only - http://bmjopen.bmj.com/site/about/guidelines.xhtml

advice strategy, and the effect of the dietary advice on the microbiota composition and on central responses to food-cues in brain areas involved in appetite regulation.

In summary, this randomized controlled trial will demonstrate the effectiveness of personalized dietary advice aiming at increasing protein intake to at least 1.2 g protein/kg BW/d on physical functioning in older adults with a lower habitual protein intake, with or without the advice to consume protein in close proximity of any usual physical activity.

For peer review only

For peer review only - http://bmjopen.bmj.com/site/about/guidelines.xhtml

List of abbreviations

aBW = adjusted body weight

BMI = body mass index

BW = body weight

fMRI-scan: Functional Magnetic Resonance Imaging scan

MMSE = Mini Mental State Examination

MPS = muscle protein synthesis

PROMISS = PRevention Of Malnutrition In Senior Subjects

QoL = quality of life

RCT = randomized controlled trial

RDA = recommended daily allowance

RUD = Resource Utilization in Dementia Questionnaire

SARC-F = Simple Questionnaire to Rapidly Diagnose Sarcopenia

SD = standard deviation

SPPB = Short Physical Performance Battery

For peer review only

Declarations

Ethics approval and consent to participate

The study has been approved by the Ethics Committee of the Helsinki University Central Hospital, Finland and The Medical Ethical Committee of the Amsterdam UMC, location VUmc, Amsterdam, the Netherlands. Oral informed consent will be obtained from each participants before the screening procedure and written informed (please see appendix I) consent will be obtained from each participant before any measurement take place.

Consent for publication

Not applicable. Personal data were not identifiable during the analysis.

Availability of data and materials

Datasets from this research will be stored at the repository of the Vrije Universiteit Amsterdam, the Netherlands and potentially available for other researchers after submitting a research proposal.

Competing interests

The authors declare that they have no competing interests.

Funding

Funding for this research is provided by EU Horizon 2020 PROMISS Project 'Prevention Of Malnutrition In Senior Subjects in the EU', Grant agreement no. 678732. Funding sponsors did not participate in the study design, collection, management, analysis and interpretation of

For peer review only

data; or writing of the manuscript. They did not participate in the decision to submit the report for publication, nor had ultimate authority over any of these activities.

Protein enriched food products

Protein enriched food products are provided by Kellogg and Fonterra. Costs for these products are also funded through the EU Horizon 2020 PROMISS grant.

Author Contributions

IR, *HAHW*, *IAB*, *MRO* and *MV* obtained funding for the PROMISS project. *IR* and *HAHW* coordinate the trial center at the Vrije Universiteit Amsterdam, the Netherlands. *SKJ* and *MHS* coordinate the trial center at the Helsinki University, Finland. All authors contributed to conception of and designing the trial. *IR* drafted the manuscript. *JB* provided cost-effectiveness expertise in clinical trial design. *LDK* provided statistical expertise and will conduct the primary statistical analysis. KSF drafted the sections for the microbiota and fMRI ancillary studies. *MCAK* and *LML* drafted the section for the persuasive technology study. *HAHW*, *SKJ*, *MHS*, *RN*, *IAB*, *MRO*, *KHP*, *RV* and *MV* critically reviewed the manuscript. All authors approved the final version.

Acknowledgements

We acknowlegde the members of the PROMISS trial group (please see Appendix II) and we thank the study participants.

Trial status

The Trial was registered in ClinicalTrials.gov. Title of registration: The (Cost)Effectiveness of Increasing Protein Intake on Physical Functioning in Older Adults. Number of

For peer review only

identification: NCT03712306. October 2018. Recruitment commenced in November 2018 and ended in November 2019.

For peer review only

References

1. EFSA. Scientific Opinion on Dietary Reference Values for protein. EFSA Panel on Dietetic Products, Nutrition and Allergies (NDA). EFSA Journal. 2012;10:2257.

2. Bauer J, Biolo G, Cederholm T, Cesari M, Cruz-Jentoft AJ, Morley JE, et al. Evidence-based recommendations for optimal dietary protein intake in older people: a position paper from the PROT-AGE Study Group. J Am Med Dir Assoc. 2013;14:542-559.

3. Deutz NE, Bauer JM, Barazzoni R, Biolo G, Boirie Y, Bosy-Westphal A, et al. Protein intake and exercise for optimal muscle function with aging: recommendations from the ESPEN Expert Group. Clin Nutr. 2014;33:929-936.

4. Cuthbertson D, Smith K, Babraj J, Leese G, Waddell T, Atherton P, et al. Anabolic signaling deficits underlie amino acid resistance of wasting, aging muscle. FASEB J. 2005;19:422-424.

5. Moore DR, Churchward-Venne TA, Witard O, Breen L, Burd NA, Tipton KD, et al. Protein ingestion to stimulate myofibrillar protein synthesis requires greater relative protein intakes in healthy older versus younger men. J Gerontol A Biol Sci Med Sci. 2015;70:57-62.

6. Wall BT, Gorissen SH, Pennings B, Koopman R, Groen BB, Verdijk LB, et al. Aging Is Accompanied by a Blunted Muscle Protein Synthetic Response to Protein Ingestion. PLoS One. 2015;10:e0140903.

7. Murphy CH, Saddler NI, Devries MC, McGlory C, Baker SK, Phillips SM. Leucine supplementation enhances integrative myofibrillar protein synthesis in free-living older men consuming lower- and higher-protein diets: a parallel-group crossover study. Am J Clin Nutr. 2016;104:1594-1606.

For peer review only

8. Holwerda AM, Paulussen KJM, Overkamp M, Goessens JPB, Kramer IF, Wodzig W, et al. Dose-Dependent Increases in Whole-Body Net Protein Balance and Dietary Protein-Derived Amino Acid Incorporation into Myofibrillar Protein During Recovery from Resistance Exercise in Older Men. J Nutr. 2019;149:221-230.

9. Houston DK, Nicklas BJ, Ding J, Harris TB, Tylavsky FA, Newman AB, et al. Dietary protein intake is associated with lean mass change in older, community-dwelling adults: the Health, Aging, and Body Composition (Health ABC) Study. Am J Clin Nutr. 2008;87:150-155.

10. Houston DK, Tooze JA, Garcia K, Visser M, Rubin S, Harris TB, et al. Protein Intake and Mobility Limitation in Community-Dwelling Older Adults: the Health ABC Study. J Am Geriatr Soc. 2017;65:1705-1711.

11. Gray-Donald K, St-Arnaud-McKenzie D, Gaudreau P, Morais JA, Shatenstein B, Payette H. Protein intake protects against weight loss in healthy community-dwelling older adults. J Nutr. 2014;144:321-326.

12. Mendonca N, Granic A, Hill TR, Siervo M, Mathers JC, Kingston A, et al. Protein Intake and Disability Trajectories in Very Old Adults: The Newcastle 85+ Study. J Am Geriatr Soc. 2019;67:50-56.

13. Ten Haaf DSM, Nuijten MAH, Maessen MFH, Horstman AMH, Eijsvogels TMH, Hopman MTE. Effects of protein supplementation on lean body mass, muscle strength, and physical performance in nonfrail community-dwelling older adults: a systematic review and meta-analysis. Am J Clin Nutr. 2018;108:1043-1059.

14. Layman DK. Dietary Guidelines should reflect new understandings about adult protein needs. Nutr Metab (Lond). 2009;6:12.

15. Paddon-Jones D, Campbell WW, Jacques PF, Kritchevsky SB, Moore LL, Rodriguez NR, et al. Protein and healthy aging. Am J Clin Nutr. 2015;

For peer review only

16. Park Y, Choi JE, Hwang HS. Protein supplementation improves muscle mass and physical performance in undernourished prefrail and frail elderly subjects: a randomized, double-blind, placebo-controlled trial. Am J Clin Nutr. 2018;108:1026-1033.

17. Ten Haaf DSM, Eijsvogels TMH, Bongers C, Horstman AMH, Timmers S, de Groot L, et al. Protein supplementation improves lean body mass in physically active older adults: a randomized placebo-controlled trial. J Cachexia Sarcopenia Muscle. 2019;

18. Beelen J, de Roos NM, de Groot L. A 12-week intervention with protein-enriched foods and drinks improved protein intake but not physical performance of older patients during the first 6 months after hospital release: a randomised controlled trial. Br J Nutr. 2017;117:1541-1549.

19. Bhasin S, Apovian CM, Travison TG, Pencina K, Moore LL, Huang G, et al. Effect of Protein Intake on Lean Body Mass in Functionally Limited Older Men: A Randomized Clinical Trial. JAMA Intern Med. 2018;178:530-541.

20. Cermak NM, Res PT, de Groot LC, Saris WH, van Loon LJ. Protein supplementation augments the adaptive response of skeletal muscle to resistance-type exercise training: a meta-analysis. Am J Clin Nutr. 2012;96:1454-1464.

21. Finger D, Goltz FR, Umpierre D, Meyer E, Rosa LH, Schneider CD. Effects of protein supplementation in older adults undergoing resistance training: a systematic review and meta-analysis. Sports Med. 2015;45:245-255.

22. Rondanelli M, Klersy C, Terracol G, Talluri J, Maugeri R, Guido D, et al. Whey protein, amino acids, and vitamin D supplementation with physical activity increases fat-free mass and strength, functionality, and quality of life and decreases inflammation in sarcopenic elderly. Am J Clin Nutr. 2016;103:830-840.

For peer review only

23. Liao CD, Tsauo JY, Wu YT, Cheng CP, Chen HC, Huang YC, et al. Effects of protein supplementation combined with resistance exercise on body composition and physical function in older adults: a systematic review and meta-analysis. Am J Clin Nutr. 2017;106:1078-1091.

24. Englund DA, Kirn DR, Koochek A, Zhu H, Travison TG, Reid KF, et al. Nutritional Supplementation With Physical Activity Improves Muscle Composition in Mobility-Limited Older Adults, The VIVE2 Study: A Randomized, Double-Blind, Placebo-Controlled Trial. J Gerontol A Biol Sci Med Sci. 2017;73:95-101.

25. Mori H, Tokuda Y. Effect of whey protein supplementation after resistance exercise on the muscle mass and physical function of healthy older women: A randomized controlled trial. Geriatr Gerontol Int. 2018;18:1398-1404.

26. Seino S, Sumi K, Narita M, Yokoyama Y, Ashida K, Kitamura A, et al. Effects of Low-Dose Dairy Protein Plus Micronutrient Supplementation during Resistance Exercise on Muscle Mass and Physical Performance in Older Adults: A Randomized, Controlled Trial. J Nutr Health Aging. 2018;22:59-67.

27. Cribb PJ, Hayes A. Effects of supplement timing and resistance exercise on skeletal muscle hypertrophy. Med Sci Sports Exerc. 2006;38:1918-1925.

28. Cederholm T, Jensen GL, Correia M, Gonzalez MC, Fukushima R, Higashiguchi T, et al. GLIM criteria for the diagnosis of malnutrition - A consensus report from the global clinical nutrition community. Clin Nutr. 2019;38:1-9.

29. Fouque D, Aparicio M. Eleven reasons to control the protein intake of patients with chronic kidney disease. Nat Clin Pract Nephrol. 2007;3:383-392.

30. Fouque D, Pelletier S, Mafra D, Chauveau P. Nutrition and chronic kidney disease. Kidney Int. 2011;80:348-357.

For peer review only

For peer review only - http://bmjopen.bmj.com/site/about/guidelines.xhtml

820 31. Eyre S, Attman PO, Haraldsson B. Positive effects of protein restriction in patients with chronic kidney disease. J Ren Nutr. 2008;18:269-280.

822 32. Folstein MF, Folstein SE, McHugh PR. "Mini-mental state". A practical method for grading the cognitive state of patients for the clinician. J Psychiatr Res. 1975;12:189-198.

825 33. Berner LA, Becker G, Wise M, Doi J. Characterization of dietary protein among older adults in the United States: amount, animal sources, and meal patterns. J Acad Nutr Diet. 2013;113:809-815.

828 34. Wijnhoven HAH, Elstgeest LEM, de Vet HCW, Nicolaou M, Snijder MB, Visser M. Development and validation of a short food questionnaire to screen for low protein intake in community-dwelling older adults: The Protein Screener 55+ (Pro55+). PLoS One. 2018;13:e0196406.

832 35. The National Institute for Health and Welfare. Fineli: the National Food Composition Database in Finland. [Available from: https://fineli.fi/fineli/en/index?

834 36. Nederlands Voedingsstoffenbestand 2016 (Dutch Food Composition Table 2016): Voedingscentrum 2016; [Available from: https://nevo-online.rivm.nl/.

836 37. English KL, Paddon-Jones D. Protecting muscle mass and function in older adults during bed rest. Curr Opin Clin Nutr Metab Care. 2010;13:34-39.

838 38. Paddon-Jones D, Rasmussen BB. Dietary protein recommendations and the prevention of sarcopenia. Curr Opin Clin Nutr Metab Care. 2009;12:86-90.

840 39. Deutz NE, Wolfe RR. Is there a maximal anabolic response to protein intake with a meal? Clin Nutr. 2013;32:309-313.

842 40. Nabuco HCG, Tomeleri CM, Sugihara Junior P, Fernandes RR, Cavalcante EF, Antunes M, et al. Effects of Whey Protein Supplementation Pre- or Post-Resistance

For peer review only - http://bmjopen.bmj.com/site/about/guidelines.xhtml

Training on Muscle Mass, Muscular Strength, and Functional Capacity in Pre-Conditioned Older Women: A Randomized Clinical Trial. Nutrients. 2018;10:

41. Simonsick EM, Montgomery PS, Newman AB, Bauer DC, Harris T. Measuring fitness in healthy older adults: the Health ABC Long Distance Corridor Walk. J Am Geriatr Soc. 2001;49:1544-1548.

42. Simonsick EM, Fan E, Fleg JL. Estimating cardiorespiratory fitness in well-functioning older adults: treadmill validation of the long distance corridor walk. J Am Geriatr Soc. 2006;54:127-132.

43. Newman AB, Simonsick EM, Naydeck BL, Boudreau RM, Kritchevsky SB, Nevitt MC, et al. Association of long-distance corridor walk performance with mortality, cardiovascular disease, mobility limitation, and disability. JAMA. 2006;295:2018-2026.

44. Beaudart C, Rolland Y, Cruz-Jentoft AJ, Bauer JM, Sieber C, Cooper C, et al. Assessment of Muscle Function and Physical Performance in Daily Clinical Practice : A position paper endorsed by the European Society for Clinical and Economic Aspects of Osteoporosis, Osteoarthritis and Musculoskeletal Diseases (ESCEO). Calcif Tissue Int. 2019;105:1-14.

45. Sayers SP, Guralnik JM, Newman AB, Brach JS, Fielding RA. Concordance and discordance between two measures of lower extremity function: 400 meter self-paced walk and SPPB. Aging Clin Exp Res. 2006;18:100-106.

46. Guralnik JM, Simonsick EM, Ferrucci L, Glynn RJ, Berkman LF, Blazer DG, et al. A short physical performance battery assessing lower extremity function: association with self-reported disability and prediction of mortality and nursing home admission. J Gerontol. 1994;49:M85-94.

For peer review only

47. Rantanen T, Era P, Heikkinen E. Maximal Isometric Muscle Strength and Socioeconomic Status, Health, and Physical Activity in 75-Year-Old Persons. Journal of Aging and Physical Activity. 1994;2:206-220.

48. Rijk JM, Roos PR, Deckx L, van den Akker M, Buntinx F. Prognostic value of handgrip strength in people aged 60 years and older: A systematic review and meta-analysis. Geriatr Gerontol Int. 2016;16:5-20.

49. Wu Y, Wang W, Liu T, Zhang D. Association of Grip Strength With Risk of All-Cause Mortality, Cardiovascular Diseases, and Cancer in Community-Dwelling Populations: A Meta-analysis of Prospective Cohort Studies. J Am Med Dir Assoc. 2017;18:551 e517-551 e535.

50. Roberts HC, Denison HJ, Martin HJ, Patel HP, Syddall H, Cooper C, et al. A review of the measurement of grip strength in clinical and epidemiological studies: towards a standardised approach. Age Ageing. 2011;40:423-429.

51. Visser M, Goodpaster BH, Kritchevsky SB, Newman AB, Nevitt M, Rubin SM, et al. Muscle mass, muscle strength, and muscle fat infiltration as predictors of incident mobility limitations in well-functioning older persons. J Gerontol A Biol Sci Med Sci. 2005;60:324-333.

52. Reinders I, Murphy RA, Koster A, Brouwer IA, Visser M, Garcia ME, et al. Muscle Quality and Muscle Fat Infiltration in Relation to Incident Mobility Disability and Gait Speed Decline: the Age, Gene/Environment Susceptibility-Reykjavik Study. J Gerontol A Biol Sci Med Sci. 2015;70:1030-1036.

53. Reinders I, Murphy RA, Brouwer IA, Visser M, Launer L, Siggeirsdottir K, et al. Muscle Quality and Myosteatosis: Novel Associations With Mortality Risk: The Age, Gene/Environment Susceptibility (AGES)-Reykjavik Study. Am J Epidemiol. 2016;183:53-60.

For peer review only

54. Volaklis KA, Halle M, Meisinger C. Muscular strength as a strong predictor of mortality: A narrative review. Eur J Intern Med. 2015;26:303-310.

55. Garcia-Hermoso A, Cavero-Redondo I, Ramirez-Velez R, Ruiz JR, Ortega FB, Lee DC, et al. Muscular Strength as a Predictor of All-Cause Mortality in an Apparently Healthy Population: A Systematic Review and Meta-Analysis of Data From Approximately 2 Million Men and Women. Arch Phys Med Rehabil. 2018;99:2100-2113 e2105.

56. Gerrits HL, Hopman MT, Sargeant AJ, de Haan A. Reproducibility of contractile properties of the human paralysed and non-paralysed quadriceps muscle. Clin Physiol. 2001;21:105-113.

57. Fried LP, Tangen CM, Walston J, Newman AB, Hirsch C, Gottdiener J, et al. Frailty in older adults: evidence for a phenotype. J Gerontol A Biol Sci Med Sci. 2001;56:M146-156.

58. Radloff LS. The CES-D Scale: A Self-Report Depression Scale for Research in the General Population. Appl Psychol Meas. 1977;1:385-401.

59. Malmstrom TK, Morley JE. SARC-F: a simple questionnaire to rapidly diagnose sarcopenia. J Am Med Dir Assoc. 2013;14:531-532.

60. Kyle UG, Genton L, Karsegard L, Slosman DO, Pichard C. Single prediction equation for bioelectrical impedance analysis in adults aged 20--94 years. Nutrition. 2001;17:248-253.

61. Sergi G, De Rui M, Veronese N, Bolzetta F, Berton L, Carraro S, et al. Assessing appendicular skeletal muscle mass with bioelectrical impedance analysis in free-living Caucasian older adults. Clin Nutr. 2015;34:667-673.

For peer review only

916 62. McCrory MA, Gomez TD, Bernauer EM, Mole PA. Evaluation of a new air displacement plethysmograph for measuring human body composition. Med Sci Sports Exerc. 1995;27:1686-1691.

919 63. EuroQol G. EuroQol--a new facility for the measurement of health-related quality of life. Health Policy. 1990;16:199-208.

921 64. Wimo A. Evaluation of the resource utilization and caregiver time in Anti-dementia drug trials-a quantitative battery. The health economics of dementia. 1998;

923 65. Wilson MM, Thomas DR, Rubenstein LZ, Chibnall JT, Anderson S, Baxi A, et al. Appetite assessment: simple appetite questionnaire predicts weight loss in community-dwelling adults and nursing home residents. Am J Clin Nutr. 2005;82:1074-1081.

927 66. Perera S, Studenski S, Newman A, Simonsick E, Harris T, Schwartz A, et al. Are estimates of meaningful decline in mobility performance consistent among clinically important subgroups? (Health ABC study). J Gerontol A Biol Sci Med Sci. 2014;69:1260-1268.

931 67. Schulz KF, Altman DG, Moher D, Group C. CONSORT 2010 Statement: updated guidelines for reporting parallel group randomised trials. BMC Med. 2010;8:18.

933 68. Lubbe LMvd, Klein MCA. Designing a system with persuasive communication to improve diet compliance for elderly users. Proceedings of the 13th EAI International Conference on Pervasive Computing Technologies for Healthcare; Trento, Italy. 3329217: ACM; 2019. p. 234-241.

937 69. Sender R, Fuchs S, Milo R. Are We Really Vastly Outnumbered? Revisiting the Ratio of Bacterial to Host Cells in Humans. Cell. 2016;164:337-340.

939 70. Schroeder BO, Backhed F. Signals from the gut microbiota to distant organs in physiology and disease. Nat Med. 2016;22:1079-1089.

For peer review only

71. Biagi E, Nylund L, Candela M, Ostan R, Bucci L, Pini E, et al. Through ageing, and beyond: gut microbiota and inflammatory status in seniors and centenarians. PLoS One. 2010;5:e10667.

72. Odamaki T, Kato K, Sugahara H, Hashikura N, Takahashi S, Xiao JZ, et al. Age-related changes in gut microbiota composition from newborn to centenarian: a cross-sectional study. BMC Microbiol. 2016;16:90.

73. David LA, Maurice CF, Carmody RN, Gootenberg DB, Button JE, Wolfe BE, et al. Diet rapidly and reproducibly alters the human gut microbiome. Nature. 2014;505:559-563.

74. Yamashita Y, Takeshita T. The oral microbiome and human health. J Oral Sci. 2017;59:201-206.

75. Testa M, Erbiti S, Delgado A, Cardenas IL. Evaluation of oral microbiota in undernourished and eutrophic children using checkerboard DNA-DNA hybridization. Anaerobe. 2016;42:55-59.

76. Deschasaux M, Bouter KE, Prodan A, Levin E, Groen AK, Herrema H, et al. Depicting the composition of gut microbiota in a population with varied ethnic origins but shared geography. Nat Med. 2018;24:1526-1531.

77. Bosch A, Levin E, van Houten MA, Hasrat R, Kalkman G, Biesbroek G, et al. Development of Upper Respiratory Tract Microbiota in Infancy is Affected by Mode of Delivery. EBioMedicine. 2016;9:336-345.

78. Mollahosseini M, Shab-Bidar S, Rahimi MH, Djafarian K. Effect of whey protein supplementation on long and short term appetite: A meta-analysis of randomized controlled trials. Clinical nutrition ESPEN. 2017;20:34-40.

79. van de Wouw M, Schellekens H, Dinan TG, Cryan JF. Microbiota-Gut-Brain Axis: Modulator of Host Metabolism and Appetite. J Nutr. 2017;147:727-745.

For peer review only

For peer review only - http://bmjopen.bmj.com/site/about/guidelines.xhtml

80. van Bloemendaal L, RG IJ, Ten Kulve JS, Barkhof F, Konrad RJ, Drent ML, et al. GLP-1 receptor activation modulates appetite- and reward-related brain areas in humans. Diabetes. 2014;63:4186-4196.

81. van Bloemendaal L, Veltman DJ, Ten Kulve JS, Groot PF, Ruhe HG, Barkhof F, et al. Brain reward-system activation in response to anticipation and consumption of palatable food is altered by glucagon-like peptide-1 receptor activation in humans. Diabetes Obes Metab. 2015;17:878-886.

82. Nutrition reference intake values for the German Speaking Countries D-A-CH ed 2, update 1 Bonn2016 [Available from: https://www.dge.de/wissenschaft/referenzwerte/protein/.

83. Nordic Council of Ministers. Nordic nutrition recommendations 2012. Integrating nutrition physical activity; 2014. pp. 1e627. Copenhagen.

84. Tieland M, Franssen R, Dullemeijer C, van Dronkelaar C, Kyung Kim H, Ispoglou T, et al. The Impact of Dietary Protein or Amino Acid Supplementation on Muscle Mass and Strength in Elderly People: Individual Participant Data and Meta-Analysis of RCT's. J Nutr Health Aging. 2017;21:994-1001.

85. Reinders I, Volkert D, de Groot L, Beck AM, Feldblum I, Jobse I, et al. Effectiveness of nutritional interventions in older adults at risk of malnutrition across different health care settings: Pooled analyses of individual participant data from nine randomized controlled trials. Clin Nutr. 2019;38:1797-1806.

86. Saunders JB, Aasland OG, Babor TF, de la Fuente JR, Grant M. Development of the Alcohol Use Disorders Identification Test (AUDIT): WHO Collaborative Project on Early Detection of Persons with Harmful Alcohol Consumption--II. Addiction. 1993;88:791-804.

For peer review only

Tables

Table 1. Eligibility criteria for participation of the PROMISS trial

**Inclusion criteria**
Community-dwelling
Age ≥ 65 years
Habitual protein intake < 1.0 g protein/kg aBW/d
BMI ≥ 18.5 kg/m^2 and ≤ 32.0 kg/m^2 (based on measured weight and height)
Self-reported ability to eat independently
Ability to speak, write and read the local language (Finnish or Dutch)
Ability to walk 400 meters within 15 minutes, without the use of a walker and no rest longer than 60 seconds
**Exclusion criteria**
Inability or unwillingness to provide informed consent
Current participation in supervised behavioural or lifestyle intervention that intervenes with the PROMISS trial
Not able the visit the research site in the following next 6 months
Bedridden, wheelchair bound or always being inside
Self-reported Parkinson's disease
Diagnosis of severe kidney disease (self-reported)
Diagnosis of type I diabetes mellitus (self-reported)
Diagnosis of type II diabetes mellitus and requiring use of insulin started within 6 months (self-reported)
Current treatment of cancer (with the exception of basal cell carcinoma)
Vegan diet

For peer review only

Severe allergies to certain food product (peanut, gluten)

Diagnosis of an eating disorder (self-reported)

Purposefully lost/gained > 3 kg in the past three months

Heart problems in the past three months, defined as heart attack, angioplasty, heart surgery, stroke, severe shortness of breath during physical activity (self-reported)

Alcohol abuse during past 6 months, defined as the AUDIT-C score ≥ 3 (86)

Low cognitive status, defined as the MMSE score ≤ 20 (32)

Abbreviations: aBW; adjusted body weight, BMI; Body Mass Index, MMSE; Mini-Mental State Examination.

For peer review only

Table 2. Measurements and time of measurements in the PROMISS trial

		**Timeline**				
		**Screening** (phone)	**Screening visit** (prior baseline)	**Baseline visit**	**3-month FU visit**	**6-month FU visit**
**Topic**	**Specific variables**					
Oral informed consent (phone)		✓				
Screening questionnaire (phone)	Sex, age, self-reported weight, height, eligibility criteria	✓				
Protein intake	Pro55+ screening (34) (phone)	✓				
Protein intake	Combination of three food diaries and three 24-h dietary recalls		✓		✓	✓
Written informed consent			✓			
Cognitive function	MMSE (32)		✓			

For peer review only - http://bmjopen.bmj.com/site/about/guidelines.xhtml

Physical functioning	400-m walk test (41, 42)	✓			✓
Antropometrics	Measured body height	✓			
Antropometrics	Measured body weight	✓		✓	✓
Demographic	Education, household		✓		
General characteristics	Perceived health, smoking status		✓		✓
Body composition	Bio-electrical impedance		✓		✓
Body composition	Air displacement plethysmography (Dutch site only)		✓		✓
Physical functioning	SPPB (46)		✓		✓
Muscle strength	Handgrip strength		✓		✓
Muscle strength	Leg extension strength		✓		✓
Self-reported mobility limitations	Ability to walk 400m and climb one flight of stairs		✓	✓	✓
Risk of sarcopenia	SARC-F questionnaire (59)		✓	✓	✓

For peer review only

Malnutrition	GLIM phenotypic criteria (28)	✓		✓
Frailty	Frailty Fried Frailty Index (57)	✓		✓
Quality of Life	EuroQol 5D (63)	✓	✓	✓
Health care costs	RUD (64)	✓	✓	✓
Appetite	SNAQ-Appetite (65)	✓		✓
Physical activity	Accelerometers	✓	✓	✓
Process evaluation				✓
Persuasive technology study				
Communication style preferences	Personality traits form	✓		
Usage data of technology	Practical experiences, interaction data of technology (number of notifications, openings, registered food intake, games)		✓	✓
Attitude towards technology	Questionnaire			✓

For peer review only

For peer review only - http://bmjopen.bmj.com/site/about/guidelines.xhtml

Microbiota study			
Oral health	Questionnaire	✓	✓
Oral microbiota	Tongue swab (16S rRNA sequencing)	✓	✓
Gut microbiota	Fresh frozen faecal sample (16S rRNA sequencing)	✓	✓
fMRI study			
Oral microbiota	Fasted unstimulated salivary sample (16S rRNA sequencing)	✓	✓
Nutritional and microbial markers	Blood sample	✓	✓
Appetite	VAS-scores of appetite and central neural responses to food-cues measured by fMRI-scan	✓	✓

For peer review only - http://bmjopen.bmj.com/site/about/guidelines.xhtml

Figure legends

Figure 1. Study timeline of the PROMISS trial

For peer review only

For peer review only - http://bmjopen.bmj.com/site/about/guidelines.xhtml

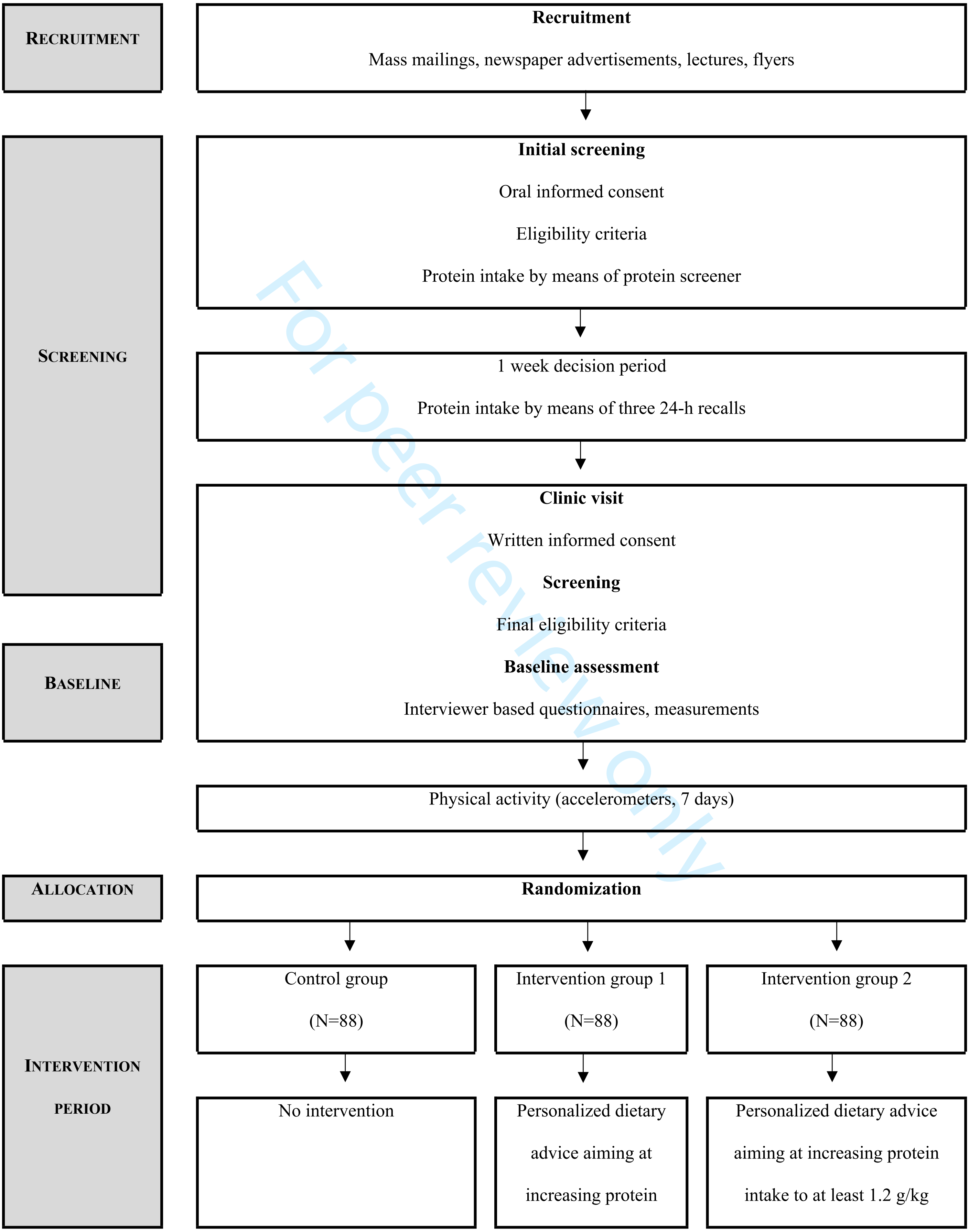

For peer review only

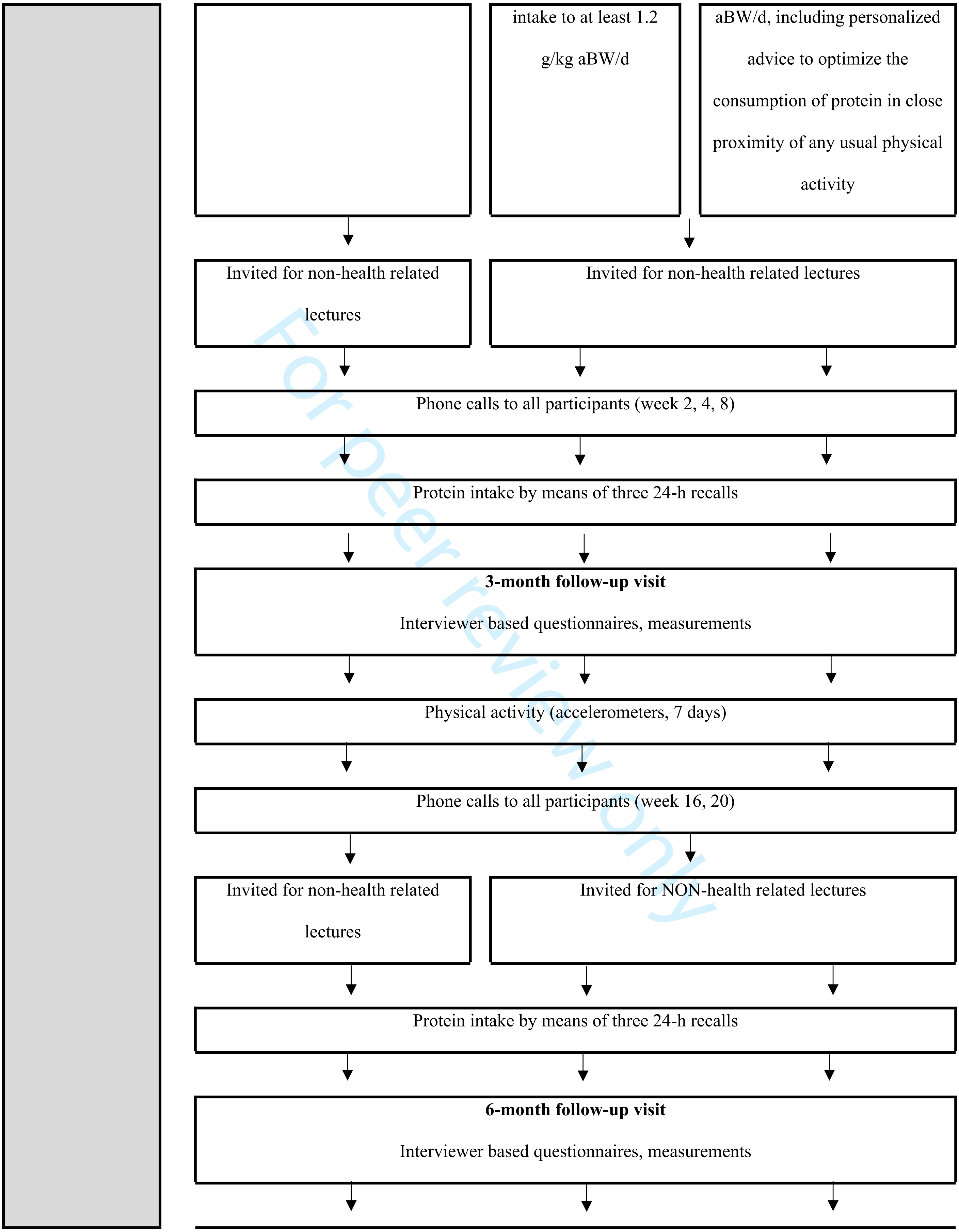

For peer review only

Physical activity (accelerometers, 7 days)

For peer review only

Toestemmingsformulier proefpersoon

Effectiviteit van het verhogen van eiwitinname op fysiek functioneren

- Ik heb de informatiebrief gelezen. Ook kon ik vragen stellen. Mijn vragen zijn voldoende beantwoord. Ik had genoeg tijd om te beslissen of ik meedoe.
- Ik weet dat meedoen vrijwillig is. Ook weet ik dat ik op ieder moment kan beslissen om toch niet mee te doen of te stoppen met het onderzoek. Daarvoor hoef ik geen reden te geven.
- Ik geef toestemming voor het informeren van mijn huisarts dat ik meedoe aan dit onderzoek.
- Ik geef toestemming voor het informeren van mijn huisarts over eventuele afwijkingen die tijdens het onderzoek gevonden worden.
- Ik geef toestemming voor het opvragen van informatie bij mijn huisarts over mijn nierfunctie, mochten er twijfels over de effecten op mijn gezondheid bestaan.
- Ik weet dat sommige mensen mijn gegevens kunnen inzien. Die mensen staan vermeld in deze informatiebrief.
- Ik geef toestemming voor het verzamelen en gebruiken van mijn gegevens op de manier en voor de doelen die in de informatiebrief staan.
- Ik geef toestemming om mijn gegevens op de onderzoekslocatie nog 15 jaar na dit onderzoek te bewaren.
- Ik wil meedoen aan dit onderzoek.

Naam proefpersoon:

Handtekening: Datum : __ / __ / __

Ik verklaar dat ik deze proefpersoon volledig heb geïnformeerd over het genoemde onderzoek.

Als er tijdens het onderzoek informatie bekend wordt die de toestemming van de proefpersoon zou kunnen beïnvloeden, dan breng ik hem/haar daarvan tijdig op de hoogte.

Naam onderzoeker (of diens vertegenwoordiger):

Handtekening: Datum: __ / __ / __

For peer review only - http://bmjopen.bmj.com/site/about/guidelines.xhtml

Appendix

Appendix II

PROMISS project coordination, Vrije Universiteit Amsterdam, Department of Health Sciences, the Netherlands

Prof. Marjolein Visser, PhD – Principle investigator of the PROMISS project

Prof. Ingeborg A Brouwer, PhD – Project manager of the PROMISS project

Margreet Olthof, PhD – Financial manager of the PROMISS project

Rachel Vijlbrief – Assistant project manager of the PROMISS project

Trial sites

Vrije Universiteit Amsterdam, the Netherlands

Hanneke Wijnhoven, PhD - local principal investigator

Nanouk Bakker Schut – research intern

Judith Bosmans, PhD – researcher

Mariska Bout – dietician and research assistant

Ingeborg Brouwer, PhD – researcher

Nona Kerremans – research intern

Lothar Kuijper, PhD – researcher

Margreet Olthof, PhD – researcher

Ilse Reinders, PhD – local co-principal investigator

Marjon Veeke – dietician and research assistant

Rachel Vijlbrief – researcher

Marjolein Visser, PhD – researcher

Merel Vrijmoeth – dietician and research assistant

For peer review only

Laura Winkens – researcher

University of Helsinki

Merja Suominen, PhD – local principal investigator

Kirsi Ali-Kovero – research assistant

Johannes Anttila – research intern

Aliisa Hyvönen – dietician and research assistant

Henriikka Jussila – research intern

Satu Jyväkorpi, PhD – local co-principal investigator

Riikka Niskanen – dietician and research assistant

Anna-Maria Piipponen – research intern and research assistant

Kaisu Pitkälä, PhD – researcher

Heli Salmenius-Suominen – researcher

Ancillary studies

Persuasive technology study

Michel Klein, PhD – principal investigator of the persuasive technology study, *Vrije Universiteit Amsterdam, the Netherlands*

Laura van der Lubbe – researcher, *Vrije Universiteit Amsterdam, the Netherlands*

Microbiota study and fMRI study

Fredrik Bäckhed, MD, PhD – researcher, *University of Gothenburg, Gothenburg, Sweden and University of Copenhagen, Copenhagen, Denmark*

Kristien Fluitman, MD – researcher, *Amsterdam UMC, location VUmc, Amsterdam, the Netherlands*

For peer review only

Richard Ijzerman, MD, PhD – researcher, *Amsterdam UMC, location VUmc, Amsterdam, the Netherlands*

Bart Keijser, PhD – researcher, *TNO earth, Zeist, the Netherlands and Academic Center for Dentistry Amsterdam, the Netherlands*

Max Nieuwdorp, MD, PhD – principal investigator of the microbiota study and fMRI study, *Amsterdam UMC, location AMC and location VUmc, Amsterdam, the Netherlands*

For peer review only

SPIRIT

STANDARD PROTOCOL ITEMS: RECOMMENDATIONS FOR INTERVENTIONAL TRIALS

SPIRIT 2013 Checklist: Recommended items to address in a clinical trial protocol and related documents*

Section/item	ItemNo	Description	Page number, section
**Administrative information**			
Title	1	Descriptive title identifying the study design, population, interventions, and, if applicable, trial acronym	1
Trial registration	2a	Trial identifier and registry name. If not yet registered, name of intended registry	4; end of abstract, 8; study design, 31; trial status
	2b	All items from the World Health Organization Trial Registration Data Set	N/A
Protocol version	3	Date and version identifier	32; trial status
Funding	4	Sources and types of financial, material, and other support	30; funding
Roles and responsibilities	5a	Names, affiliations, and roles of protocol contributors	1, 31; author contributions
	5b	Name and contact information for the trial sponsor	-
	5c	Role of study sponsor and funders, if any, in study design; collection, management, analysis, and interpretation of data; writing of the report; and the decision to submit the report for publication, including whether they will have ultimate authority over any of these activities	30, 31; funding
	5d	Composition, roles, and responsibilities of the coordinating centre, steering committee, endpoint adjudication committee, data management team, and other individuals or groups overseeing the trial, if applicable (see Item 21a for data monitoring committee)	Appendix II
**Introduction**			

For peer review only

Background and rationale	6a	Description of research question and justification for undertaking the trial, including summary of relevant studies (published and unpublished) examining benefits and harms for each intervention	5, 6, 7, 8
	6b	Explanation for choice of comparators	7
Objectives	7	Specific objectives or hypotheses	7; second paragraph
Trial design	8	Description of trial design including type of trial (eg, parallel group, crossover, factorial, single group), allocation ratio, and framework (eg, superiority, equivalence, noninferiority, exploratory)	8; study design
**Methods: Participants, interventions, and outcomes**			
Study setting	9	Description of study settings (eg, community clinic, academic hospital) and list of countries where data will be collected. Reference to where list of study sites can be obtained	8; eligibility criteria, 9-11; recruitment and screening
Eligibility criteria	10	Inclusion and exclusion criteria for participants. If applicable, eligibility criteria for study centres and individuals who will perform the interventions (eg, surgeons, psychotherapists)	8; eligibility criteria
Interventions	11a	Interventions for each group with sufficient detail to allow replication, including how and when they will be administered	13-15; intervention
	11b	Criteria for discontinuing or modifying allocated interventions for a given trial participant (eg, drug dose change in response to harms, participant request, or improving/worsening disease)	-
	11c	Strategies to improve adherence to intervention protocols, and any procedures for monitoring adherence (eg, drug tablet return, laboratory tests)	14; compliance
	11d	Relevant concomitant care and interventions that are permitted or prohibited during the trial	-
Outcomes	12	Primary, secondary, and other outcomes, including the specific measurement variable (eg, systolic blood pressure), analysis metric (eg, change from baseline, final value, time to event), method of aggregation (eg, median, proportion), and time point for each outcome. Explanation of the clinical relevance of chosen efficacy and harm outcomes is strongly recommended	16; primary outcome, 17-19; secondary outcomes, 19, 20; other measures

For peer review only - http://bmjopen.bmj.com/site/about/guidelines.xhtml

Participant timeline	13	Time schedule of enrolment, interventions (including any run-ins and washouts), assessments, and visits for participants. A schematic diagram is highly recommended (see Figure)	12, 13; study timeline, Figure 1
Sample size	14	Estimated number of participants needed to achieve study objectives and how it was determined, including clinical and statistical assumptions supporting any sample size calculations	20; sample size
Recruitment	15	Strategies for achieving adequate participant enrolment to reach target sample size	9-11; recruitment and screening
**Methods: Assignment of interventions (for controlled trials)**			
Allocation:			
Sequence generation	16a	Method of generating the allocation sequence (eg, computer-generated random numbers), and list of any factors for stratification. To reduce predictability of a random sequence, details of any planned restriction (eg, blocking) should be provided in a separate document that is unavailable to those who enrol participants or assign interventions	11, 12 Randomization, allocation and masking
Allocation concealment mechanism	16b	Mechanism of implementing the allocation sequence (eg, central telephone; sequentially numbered, opaque, sealed envelopes), describing any steps to conceal the sequence until interventions are assigned	-
Implementation	16c	Who will generate the allocation sequence, who will enrol participants, and who will assign participants to interventions	12; second paragraph of study time line
Blinding (masking)	17a	Who will be blinded after assignment to interventions (eg, trial participants, care providers, outcome assessors, data analysts), and how	12; Randomization, allocation and masking, 20; statistical analyses plan
	17b	If blinded, circumstances under which unblinding is permissible, and procedure for revealing a participant's allocated intervention during the trial	-
**Methods: Data collection, management, and analysis**			

Data collection methods	18a	Plans for assessment and collection of outcome, baseline, and other trial data, including any related processes to promote data quality (eg, duplicate measurements, training of assessors) and a description of study instruments (eg, questionnaires, laboratory tests) along with their reliability and validity, if known. Reference to where data collection forms can be found, if not in the protocol	12, 13; study timeline, 15; intervention fidelity, 16; primary outcome, 17, 18, 19; secondary outcomes, 19, 20; other measures
	18b	Plans to promote participant retention and complete follow-up, including list of any outcome data to be collected for participants who discontinue or deviate from intervention protocols	-
Data management	19	Plans for data entry, coding, security, and storage, including any related processes to promote data quality (eg, double data entry; range checks for data values). Reference to where details of data management procedures can be found, if not in the protocol	22; Data quality assurance and data management
Statistical methods	20a	Statistical methods for analysing primary and secondary outcomes. Reference to where other details of the statistical analysis plan can be found, if not in the protocol	20, 21, 22; Statistical analyses plan
	20b	Methods for any additional analyses (eg, subgroup and adjusted analyses)	20, 21, 22; Statistical analyses plan
	20c	Definition of analysis population relating to protocol non-adherence (eg, as randomised analysis), and any statistical methods to handle missing data (eg, multiple imputation)	20, 21, 22; Statistical analyses plan
**Methods: Monitoring**			
Data monitoring	21a	Composition of data monitoring committee (DMC); summary of its role and reporting structure; statement of whether it is independent from the sponsor and competing interests; and reference to where further details about its charter can be found, if not in the protocol. Alternatively, an explanation of why a DMC is not needed	-
	21b	Description of any interim analyses and stopping guidelines, including who will have access to these interim results and make the final decision to terminate the trial	-

Harms	22	Plans for collecting, assessing, reporting, and managing solicited and spontaneously reported adverse events and other unintended effects of trial interventions or trial conduct	22; Participants' safety
Auditing	23	Frequency and procedures for auditing trial conduct, if any, and whether the process will be independent from investigators and the sponsor	-
**Ethics and dissemination**			
Research ethics approval	24	Plans for seeking research ethics committee/institutional review board (REC/IRB) approval	30; Ethics approval and consent to participate
Protocol amendments	25	Plans for communicating important protocol modifications (eg, changes to eligibility criteria, outcomes, analyses) to relevant parties (eg, investigators, REC/IRBs, trial participants, trial registries, journals, regulators)	-
Consent or assent	26a	Who will obtain informed consent or assent from potential trial participants or authorised surrogates, and how (see Item 32)	9, 10, 11 recruitment and screening, 12, 13; study timeline, 30; Ethics approval and consent to participate
	26b	Additional consent provisions for collection and use of participant data and biological specimens in ancillary studies, if applicable	23, 24; Persuasive technology study, 24, 25, 26; Microbiota study, 26; fMRI study.
Confidentiality	27	How personal information about potential and enrolled participants will be collected, shared, and maintained in order to protect confidentiality before, during, and after the trial	22, 23; Data quality assurance and data management, 30; Consent for publication
Declaration of interests	28	Financial and other competing interests for principal investigators for the overall trial and each study site	30; Competing interests
Access to data	29	Statement of who will have access to the final trial dataset, and disclosure of contractual agreements that limit such access for investigators	30; Availability of data and materials

For peer review only

Ancillary and post-trial care	30	Provisions, if any, for ancillary and post-trial care, and for compensation to those who suffer harm from trial participation	N/A
Dissemination policy	31a	Plans for investigators and sponsor to communicate trial results to participants, healthcare professionals, the public, and other relevant groups (eg, via publication, reporting in results databases, or other data sharing arrangements), including any publication restrictions	-
	31b	Authorship eligibility guidelines and any intended use of professional writers	-
	31c	Plans, if any, for granting public access to the full protocol, participant-level dataset, and statistical code	-
**Appendices**			
Informed consent materials	32	Model consent form and other related documentation given to participants and authorised surrogates	Appendix I
Biological specimens	33	Plans for collection, laboratory evaluation, and storage of biological specimens for genetic or molecular analysis in the current trial and for future use in ancillary studies, if applicable	N/A

*It is strongly recommended that this checklist be read in conjunction with the SPIRIT 2013 Explanation & Elaboration for important clarification on the items. Amendments to the protocol should be tracked and dated. The SPIRIT checklist is copyrighted by the SPIRIT Group under the Creative Commons "Attribution-NonCommercial-NoDerivs 3.0 Unported" license.

For peer review only

BMJ Open

Effectiveness and cost-effectiveness of personalized dietary advice aiming at increasing protein intake on physical functioning in community-dwelling older adults with lower habitual protein intake: rationale and design of the PROMISS randomized controlled trial

Journal:	*BMJ Open*
Manuscript ID	bmjopen-2020-040637.R1
Article Type:	Protocol
Date Submitted by the Author:	18-Sep-2020
Complete List of Authors:	Reinders, Ilse; Vrije Universiteit Amsterdam,<br>Wijnhoven, Hanneke; Vrije Universiteit Amsterdam<br>Jyvakorpi, S; University of Helsinki<br>Suominen, Merja; University of Helsinki<br>Niskanen, Riikka; University of Helsinki<br>Bosmans, J; Vrije Universiteit Amsterdam<br>Brouwer, Ingeborg; Vrije Universiteit Amsterdam<br>Fluitman, Kristien; Amsterdam UMC; University of Gothenburg<br>Klein, Michel; Vrije Universiteit Amsterdam<br>Kuijper, Lothar; Vrije Universiteit Amsterdam<br>van der Lubbe, Laura; Vrije Universiteit Amsterdam<br>Olthof, Margreet R.; Vrije Universiteit Amsterdam<br>Pitkala, Kaisu H.; University of Helsinki<br>van der Pols-Vijlbrief, Rachel ; Vrije Universiteit Amsterdam<br>Visser, Marjolein; Vrije Universiteit Amsterdam
**Primary Subject Heading**:	Nutrition and metabolism
Secondary Subject Heading:	Public health
Keywords:	GERIATRIC MEDICINE, NUTRITION & DIETETICS, PUBLIC HEALTH

SCHOLARONE™
Manuscripts

I, the Submitting Author has the right to grant and does grant on behalf of all authors of the Work (as defined in the below author licence), an exclusive licence and/or a non-exclusive licence for contributions from authors who are: i) UK Crown employees; ii) where BMJ has agreed a CC-BY licence shall apply, and/or iii) in accordance with the terms applicable for US Federal Government officers or employees acting as part of their official duties; on a worldwide, perpetual, irrevocable, royalty-free basis to BMJ Publishing Group Ltd ("BMJ") its licensees and where the relevant Journal is co-owned by BMJ to the co-owners of the Journal, to publish the Work in this journal and any other BMJ products and to exploit all rights, as set out in our licence.

The Submitting Author accepts and understands that any supply made under these terms is made by BMJ to the Submitting Author unless you are acting as an employee on behalf of your employer or a postgraduate student of an affiliated institution which is paying any applicable article publishing charge ("APC") for Open Access articles. Where the Submitting Author wishes to make the Work available on an Open Access basis (and intends to pay the relevant APC), the terms of reuse of such Open Access shall be governed by a Creative Commons licence – details of these licences and which Creative Commons licence will apply to this Work are set out in our licence referred to above.

Other than as permitted in any relevant BMJ Author's Self Archiving Policies, I confirm this Work has not been accepted for publication elsewhere, is not being considered for publication elsewhere and does not duplicate material already published. I confirm all authors consent to publication of this Work and authorise the granting of this licence.

For peer review only

Effectiveness and cost-effectiveness of personalized dietary advice aiming at increasing protein intake on physical functioning in community-dwelling older adults with lower habitual protein intake: rationale and design of the PROMISS randomized controlled trial

Ilse Reinders[1], Hanneke A.H. Wijnhoven[1], Satu K. Jyväkorpi[2], Merja H. Suominen[2], Riikka Niskanen[2], Judith E. Bosmans[1], Ingeborg A. Brouwer[1], Kristien S. Fluitman[3,4], Michel C.A. Klein[5], Lothar D. Kuijper[1], Laura M. van der Lubbe[5], Margreet R. Olthof[1], Kaisu H. Pitkälä[2], Rachel Vijlbrief[1] and Marjolein Visser[1].

[1]Department of Health Sciences, Faculty of Science, and the Amsterdam Public Health research institute, Vrije Universiteit Amsterdam, The Netherlands; [2] University of Helsinki, Department of General Practice and Primary Health Care, and Helsinki University Central Hospital, Unit of Primary Health Care, Finland; [3]Department of Internal Medicine, Amsterdam UMC, Vrije Universiteit Amsterdam, Amsterdam Public Health research institute, Amsterdam, The Netherlands; [4]Wallenburg Laboratory, Department of Molecular and Clinical Medicine, Sahlgrenska Academy, University of Gothenburg, Gothenburg, Sweden; [5]Department of Computer Science, Vrije Universiteit Amsterdam, Amsterdam, the Netherlands.

Corresponding author:
Ilse Reinders
Vrije Universiteit Amsterdam
De Boelelaan 1105, room O-526
1081 HV Amsterdam

For peer review only - http://bmjopen.bmj.com/site/about/guidelines.xhtml

The Netherlands

ilse.reinders@vu.nl

Tel: +31205982467

Total word count: 7855

For peer review only

For peer review only - http://bmjopen.bmj.com/site/about/guidelines.xhtml

Abstract

Introduction: Short-term metabolic and observational studies suggest that protein intake above the recommended dietary allowance of 0.83 g/kg body weight (BW)/d may support preservation of lean body mass and physical function in old age, but evidence from randomized controlled trials is inconclusive.

Methods and analysis: The PRevention Of Malnutrition In Senior Subjects in the EU (PROMISS) trial examines the effect of personalized dietary advice aiming at increasing protein intake with or without advice regarding timing of protein intake to close proximity of usual physical activity, on change in physical functioning after 6 months among community-dwelling older adults (≥ 65 y) with a habitual protein intake of < 1.0 g/kg adjusted (a)BW/d. Participants (n=264) will be recruited in Finland and the Netherlands, and will be randomized into three groups; two intervention groups and one control group. Intervention group 1 (n=88) receives personalized dietary advice and protein enriched food products in order to increase their protein intake to at least 1.2 g/kg aBW/d. Intervention group 2 (n=88) receives the same advice as described for intervention group 1, and in addition advice to consume 7.5-10 g protein through protein (en)rich(ed) foods within half an hour after performing usual physical activity. The control group (n=88) receives no intervention. All participants will be invited to attend lectures not related to health. The primary outcome is 6-months change in physical functioning measured by change in walk time using a 400-m walk test. Secondary outcomes are: 6-months change in the Short Physical Performance Battery score, muscle strength, body composition, self-reported mobility limitations, quality of life, incidence of frailty, incidence of sarcopenia risk and incidence of malnutrition. We also investigate cost-effectiveness by change in health care costs.

Discussion: The PROMISS trial will provide evidence whether increasing protein intake, and additionally optimizing the timing of protein intake, has a positive effect on the course of

For peer review only

For peer review only - http://bmjopen.bmj.com/site/about/guidelines.xhtml

physical functioning after 6 months among community-dwelling older adults with a habitual protein intake of < 1.0 g/kg aBW/d.

Ethics and disseminations: The study has been approved by the Ethics Committee of the Helsinki University Central Hospital, Finland (ID of the approval; HUS/1530/2018) and The Medical Ethical Committee of the Amsterdam UMC, location VUmc, Amsterdam, the Netherlands (ID of the approval; 2018.399). All participants provided written informed consent prior to being enrolled onto the study. Results will be submitted for publication in peer-reviewed journals and will be made available to stakeholders (i.e. older adults, heath care professionals and industry).

Trial status

The Trial was registered in ClinicalTrials.gov. Title of registration: The (Cost)Effectiveness of Increasing Protein Intake on Physical Functioning in Older Adults. Number of identification: NCT03712306. Registered 19 October 2018. Recruitment commenced in October 2018 and ended in November 2019.

Keywords: older adults, protein intake, physical functioning, RCT, malnutrition, protein recommendation, 400 meter walk.

For peer review only

Strength and limitations of this study

- This large randomized controlled trial addresses a key question whether dietary advice to increase protein intake to ≥ 1.2 g/kg adjusted body weight (aBW)/d is beneficial for physical functioning in community-dwelling older adults.
- This trial will also examine the additional effect of the timing of protein intake in close proximity of usual physical activity on change in physical functioning.
- Participants included had a habitual protein intake of < 1.0 g/kg aBW/d.
- The lack of blinding of the study participants and nutritionists who also collect data on all outcome measures is a limitation of the study design.
- Another limitation of this study is that the biological value of the total protein intake (i.e. type of amino acids) is unknown.

For peer review only

Introduction

There is an ongoing debate on whether or not older adults should be recommended a protein intake above the current recommended daily allowance (RDA) established by the European Food Safety Authority (EFSA) of 0.83 g/kg body weight (BW)/d for adults (1). International panels of geriatricians, nutritional experts and scientists have proposed at least 1.0-1.2 g protein/kg BW/d for healthy older adults in order to maintain and regain muscle mass, strength and function (2, 3).

The proposed increase of the RDA for older adults is merely based on results from short term metabolic and epidemiological studies. Several metabolic studies showed that older adults (≥ 65 y) have a lower muscle protein synthesis (MPS) following protein intake compared to younger adults (4-6), and that higher protein intake enhances MPS in older adults when compared to lower protein intake (1.2 g/kg BW/d vs. 0.8 g/kg BW/d (7), or ≥ 30 g/d vs. 15 g/d (8)). In addition, the anabolic threshold (i.e. optimal dose of dietary protein in a meal that stimulates MPS) is 70% higher in older compared to younger adults (5). Epidemiological studies have shown that higher dietary protein intake in older adults, defined as > 0.9 g/kg BW/d (9) or > 1.0 g/kg BW/d (10-12) is associated with lower risk of weight loss (11), better disability trajectories (12), less loss of lean mass (9), or lower risk of developing functional impairments (10).

Despite the evidence from metabolic and epidemiological studies, causal evidence to support beneficial effects of protein intake at or above 1.0 g/kg BW/d based on randomized controlled trials (RCTs) is not conclusive. One systematic review showed no beneficial effect of increasing protein intake on lean body mass, muscle cross-sectional area, muscle strength, or physical performance (13). Of the 36 studies included in the systematic review, 26 studies presented mean habitual protein intake of the study participants which ranged between 0.78 and 1.5 g/kg BW/d, with only one study below the protein RDA of 0.8 g/kg BW/d (13). The

For peer review only

relatively high mean habitual protein intake may explain the absence of a beneficial effect of additional protein. Another explanation may be that the amount of protein provided might not have been sufficient to augment MPS. I.e. a protein intake of 25 – 30 g is required to stimulate MPS and maintain muscle mass (14, 15), though the amounts provided varied between 10 g/d (3d/wk) and total intake of 125 g/day or were not reported. Of the trials published after the systematic review, Park et al. showed that intake of 1.5 g/kg BW/d for 12 weeks resulted in higher muscle mass and improved gait speed compared to intake of 0.8 g/kg BW/d in undernourished pre-frail and frail older adults (16). Ten Haaf et al. showed a positive effect of increasing protein intake for 12 weeks on lean body mass in active older adults with a habitual protein intake of < 1.0 g/kg BW/d (17). Beelen et al. found no effects of protein supplementation on physical performance among older adults after hospital discharge (18), however, baseline protein intake was already 1.0 g/kg BW/d in the control group and 1.5 g/kg BW/d in the intervention group. Finally, Bhasin et al. showed no beneficial effects other than a decrease in fat mass after a controlled diet with 1.3 g/kg BW/d of protein for 6 months compared to a control diet consisting of 0.8 g protein /kg BW/d (19) among functionally limited community-dwelling men aged ≥ 65 years. However, mean BMI of the participants was quite high (30.3 kg/m^2), which may have resulted in an overestimation of baseline protein requirements. Based on inconsistent findings, more RCTs in older adults with lower habitual protein intake are needed to determine the potential effect of increasing protein intake on physical functioning outcomes.

Previous studies among older adults showed that protein supplementation in combination with resistance exercise has more beneficial effects on body composition, muscle strength and physical function compared to resistance exercise alone (20-26). The underlying hypothesis is that protein supplementation augments the adaptive response of skeletal muscle to resistance exercise. In addition, there is evidence that timing of protein

For peer review only - http://bmjopen.bmj.com/site/about/guidelines.xhtml

intake in close proximity of physical activity stimulated MPS to greater extent than when timed at other hours during the day (27). To our knowledge, there are no RCTs investigating the effect of timing protein intake in close proximity of physical activities on physical functioning.

The PROMISS trial is designed to fill in some of the current knowledge gaps on the optimal amount of dietary protein in older community-dwelling adults and timing of protein intake in relation to physical activity. Its primary objective is to examine the effectiveness of personalized dietary advice aiming at increasing protein intake to at least 1.2 g/kg adjusted (a)BW/d on change in physical functioning after 6 months measured by change in walk time using a 400-m walk test among community-dwelling older adults with a habitual protein intake of < 1.0 g/kg aBW/d. Additionally, it examines the combined effect of personalized dietary advice aiming at increasing protein intake to at least 1.2 g/kg aBW/d and advice aiming at optimizing the timing of protein intake in close proximity of usual physical activity. The secondary objectives are to examine the effectiveness of personalized dietary advice aiming at increasing protein intake to at least 1.2 g/kg adjusted on 6-month changes in physical functioning measured by the Short Physical Performance Battery (SPPB) score, muscle strength, body composition, self-reported mobility limitations, quality of life, incidence of frailty, incidence of sarcopenia risk, and incidence of malnutrition and change in health care costs.

In three ancillary studies the following additional objectives are addressed; 1) the effect of using persuasive technology on adherence to personalized dietary advice aiming at increasing protein to at least 1.2 g/kg aBW/d, 2) the effect of personalized dietary advice aiming at increasing protein intake to at least 1.2 g/kg aBW/d on the oral and gut microbiota composition, and 3) the effect of personalized dietary advice aiming at increasing protein

For peer review only

For peer review only - http://bmjopen.bmj.com/site/about/guidelines.xhtml

intake to at least 1.2 g/kg aBW/d on central neural responses to food-cues in brain areas of interest.

Methods and analysis

Study design

The PROMISS trial is a multicentre randomized controlled trial (ClinicalTrials.gov identifier: NCT03712306) designed to examine the effectiveness of personalized dietary advice aiming at increasing protein intake and advice on optimizing the timing of protein intake in close proximity of usual physical activity on change in physical functioning after 6 months. Participants will be randomised into three groups: one control group (no intervention); intervention group 1) receiving personalized dietary advice aiming at increasing protein intake to at least 1.2 g/kg aBW/d; and intervention group 2) receiving personalized dietary advice aiming at increasing protein intake to at least 1.2 g/kg aBW/d, including personalized advice to optimize the timing of protein intake in close proximity of usual physical activity.

Eligibility criteria

The eligibility criteria are proposed to include a study group of community-dwelling older adults (65+) with a habitual protein intake < 1.0 g/kg aBW/d. Inclusion and exclusion criteria are listed in Table 1, and some are described in more detail below.

For peer review only - http://bmjopen.bmj.com/site/about/guidelines.xhtml

Table 1. Eligibility criteria for participation of the PROMISS trial

**Inclusion criteria**
Community-dwelling
Age ≥ 65 years
Habitual protein intake < 1.0 g protein/kg aBW/d
BMI ≥ 18.5 kg/m^2 and ≤ 32.0 kg/m^2 (based on measured weight and height)
Ability to walk 400 meters within 15 minutes, without the use of a walker and no rest longer than 60 seconds
**Exclusion criteria**
Inability or unwillingness to provide informed consent
Not able to eat independently (self-reported)
Not able to speak, write and read the local language (Finnish or Dutch)
Current participation in supervised behavioural or lifestyle intervention that intervenes with the PROMISS trial
Not able the visit the research site in the following next 6 months
Bedridden, wheelchair bound or always being inside
Diagnosis of severe kidney disease (self-reported)
Parkinson’s disease (self-reported)
Diagnosis of type I diabetes mellitus (self-reported)
Diagnosis of type II diabetes mellitus and requiring use of insulin started within 6 months (self-reported)
Current treatment of cancer (with the exception of basal cell carcinoma)
Vegan diet
Severe allergies to certain food product (peanut, gluten)
Diagnosis of an eating disorder (self-reported)

For peer review only

For peer review only - http://bmjopen.bmj.com/site/about/guidelines.xhtml

Purposefully lost/gained > 3 kg in the past three months

Heart problems in the past three months, defined as heart attack, angioplasty, heart surgery, stroke, severe shortness of breath during physical activity (self-reported)

Alcohol abuse during past 6 months, defined as the AUDIT-C score ≥ 3 (28)

Low cognitive status, defined as the MMSE score ≤ 20 (29)

Abbreviations: aBW; adjusted body weight, BMI; Body Mass Index, MMSE; Mini-Mental State Examination.

Older adults with a BMI of < 18.5 kg/m^2 will be excluded, because these participants are likely to be undernourished (30) and should preferably receive general nutritional care that is not provided in this trial. Those with a BMI of > 32.0 kg/m^2 will be also excluded, because a high BMI (> 30.0 kg/m^2) is associated with poorer physical function (31) and disability (32) in old age and intentional weight loss by lifestyle interventions lead to a reduced mortality risk (33). In light of this evidence, older adults with a BMI > 32.0 kg/m^2 should preferably be advised to lose weight, which is not the aim of the present study and may interfere with the study objective. Because participants of intervention group 2 will be advised to consume protein rich foods in close proximity of usual physical activity, older adults who are bedridden, wheelchair bound or do not go outside will be excluded from the trial. Older adults with a diagnosis of severe kidney disease (i.e. treatment of a nephrologist and/or protein-restricted diet, self-reported) will also be excluded as they should be advised to limit their protein intake (2, 34-36). Older adults with a low cognitive status (Mini-Mental State Examination (MMSE) score ≤ 20 (29)) will be excluded, as participants should be able to understand and follow dietary advice if randomized to one of the intervention groups.

<u>Calculation of protein intake using adjusted body weight</u>

For peer review only

For peer review only - http://bmjopen.bmj.com/site/about/guidelines.xhtml

200 To calculate habitual protein intake in g/kg aBW/d for all (potential) participants and recommended protein intake (participants in the two intervention groups), we apply adjusted BW depending on participants' age and BMI. We use adjusted body weight because underweight persons require extra protein to build muscle tissue, while in overweight persons, much 'extra weight' is adipose tissue. Protein intake in g/kg aBW/d is based on self-reported BW during screening and afterwards based on measured BW during the baseline assessment, which is further used throughout the study. For those with a BMI > 25.0 to 32.0 kg/m^2 (age ≤ 70 y) or > 27.0 to 32.0 kg/m^2 (age > 70 y) we apply aBW corresponding to a BMI of respectively 25.0 or 27.0 kg/m^2. For those with a BMI > 18.5 to < 22.0 kg/m^2 (age > 70 y) we apply aBW corresponding to a BMI of respectively 18.5 or 22.0 kg/m^2 (37). For the recommended protein intake, we apply adjusted BW which is based on baseline measured BW.

Recruitment and screening

Two hundred and sixty-four community-dwelling adults aged 65 years and older will be recruited at two study sites (metropolitan area of Finland including Helsinki, Espoo, Vantaa, Kauniainen, and Amsterdam, the Netherlands). The recruitment strategy includes mass mailing using addresses obtained from a random sample of the Finnish Population Registry (in Finland only), newspaper advertisements, media coverage, lectures, oral presentations to the target group, informing professionals working with older adults and flyers which will be distributed at locations where many community-dwelling older adults visit.

Older adults who are interested in participating will be asked to contact the local PROMISS research team (by phone or by e-mail). Thereafter, screening by phone takes place, only when verbal informed consent is given, in which the majority of the eligibility criteria will be assessed along with an explanation of the study. Only those with a lower

For peer review only

For peer review only - http://bmjopen.bmj.com/site/about/guidelines.xhtml

225 habitual protein intake (< 1.0 g/kg aBW/d) will be invited for the first clinic visit. Assessment
226 of habitual protein intake will be estimated in two steps: 1) initial screening by phone; 2) a
227 full dietary assessment based on a combination of three food diaries and three 24-h dietary
228 recalls to confirm lower habitual protein intake. Step 1, the initial screening is performed by
229 phone using the Protein Screener 55+ (Pro55+, available for use in English, Finnish and
230 Dutch: see www.proteinscreener.nl/#/). This screening tool was specifically developed and
231 validated for this purpose (38). The screening results in a probability score (0-100%) of
232 having a protein intake below 1.0 g/kg aBW/d. At a probability of > 30%, sensitivity and
233 specificity are optimally balanced (38). In the PROMISS trial we select persons with a
234 probability score varying between > 15% (when initial response rates to recruitment
235 strategies are low) and > 30% (when initial response rates are high), for the second step of
236 assessing habitual protein intake. Those who fulfill the eligibility criteria receive further
237 information on the study and a food diary with a booklet with pictures of portion sizes by post
238 to support the 24-h dietary recalls. After a minimum of one week of consideration, the
239 research staff contacts the older adults, and among those who are still willing to participate
240 the full dietary assessment will take place (step 2). These potential participants will be asked
241 to keep track of their dietary intake by filling out the provided food diary for three
242 consecutive days (three weekdays; or two weekdays and one weekend day). The booklet with
243 pictures of portion sizes that they received earlier will help them accurately filling out the
244 diary. Each day after, they will be called by a nutritionist to go through their food diary of the
245 day before (24-h dietary recall). Potential participants are asked whether these days are
246 representative for their habitual diet. In case one of the three days is not representative, mean
247 protein intake is based on two instead of three days. In case of more than one non-
248 representative day, the person will be excluded. The food intake data based on the 24-h
249 dietary recall will be entered into the program 'Fineli' for the Finnish data (39) and into the

For peer review only

program 'Eetmeter' of the Dutch Nutrition Center using an extended version of the Dutch Food Composition Table of 2016 for the Dutch data (40) to calculate intake of macronutrients and micronutrients (vitamin D and vitamin B12). Participants with an actual protein intake ≥ 1.0 g/kg aBW/d (based on self-reported BW) will be excluded.

Potential participants with a mean habitual protein intake < 1.0 g/kg aBW/d (based on the three 24-h dietary recalls), will be invited for the clinic visit, where final eligibility criteria will be assessed; MMSE > 20, ability to walk 400 m within 15 minutes (the use of a cane is allowed, but without the use of a walker and no rest longer than 60 seconds), and BMI of ≥ 18.5 kg/m^2 and ≤ 32.0 kg/m^2 based on measured BW and body height. When all eligibility criteria are met, participants are included in the PROMISS trial.

Randomization, allocation and masking

Randomization by means of a stratified block randomization procedure will be performed by an independent statistician. Participants will be allocated in a 1:1:1 ratio to the three groups. The size of the randomization blocks is three. Participants will be stratified according to their baseline habitual protein intake (< 0.9 or 0.9-1.0 g/kg aBW/d) and sex to ensure homogeneous distribution of baseline habitual protein intake and sex in the three groups across the two recruitment sites, because there may be a different intervention effect by baseline habitual protein or sex. In case couples are eligible we will allocate them to the same intervention group to limit interference between intervention groups. We will randomly select on which partner the randomization for the intervention group is based. Any resulting unbalance in the number of subjects per treatment arm will be corrected in the randomization of the next block. Due to the nature of the study, researchers, nutritionists and participants are not blinded to the study group.

For peer review only

For peer review only - http://bmjopen.bmj.com/site/about/guidelines.xhtml

Study timeline

The first clinic visit starts with written informed consent, and when participants are eligible, the baseline assessment will be performed. The baseline assessment consists of questionnaires (frailty status, risk of sarcopenia, self-reported mobility limitations, quality of life (QoL) and health care costs) and measurements (physical function, muscle strength and body composition). See below 'primary and secondary outcomes' and 'other measures' for details on these assessments. An accelerometer will be attached to measure physical activity for 7 subsequent days.

After the baseline assessment, participants will be randomised to one of the three study groups done by the nutritionists and they will inform the participants in which group they are allocated to. Participants randomized to one of the two intervention groups will be invited for a consultation meeting at the clinic to receive their personalized dietary advice, and personalized advice on optimizing the timing of protein intake in close proximity of usual physical activity (intervention group 2 only). This will take place within 2 weeks after the baseline assessment since the personalized advice needs to be composed by the nutritionist. The baseline assessment is considered the start of the study period for participants of the control group, while the consultation meeting is considered the start of the study period for participants of the intervention groups.

One week prior to the 3-month follow-up visit, dietary intake will be assessed again by means of a combination of three food diaries and three 24-h dietary recalls. The 3-month follow-up visit will take place at the clinic and includes measurement of BW, assessment of self-reported mobility limitations, risk of sarcopenia, QoL, health care costs and the accelerometer will be attached to measure physical activity for 7 subsequent days.

One week prior to the 6-month follow-up visit (final measurement) dietary intake will again be assessed by means of a combination of three food diaries and three 24-h dietary

For peer review only

recalls, which allows us to determine compliance to the dietary advice. The 6-month follow-up visit at the clinic includes all measurements performed during the baseline visit and the accelerometer is attached again to measure physical activity for the next 7 days. Finally, among participants of the intervention groups only, several questions regarding the appreciation and adherence of the intervention and participants' intention to follow the dietary advice in the future will be asked in order to perform a process-evaluation. Figure 1 shows the study timeline and Table 2 provides an overview of all measurements.

Intervention

Participants in the intervention group 1 will receive a personalized dietary advice face-to-face by nutritionists dedicated to this study aiming at increasing their protein intake to at least 1.2 g/kg aBW/d (without increasing total daily energy intake), based on their habitual dietary characteristics (based on three 24-h recalls), protein intake and measured BW assessed at baseline. Participants will be asked if they usually prepare the main meal; whether they eat the meal at a e.g. community home; whether they consume ready-to-eat meals; whether they use meal services; and if they eat at family or friends' home. All their answers will be incorporated in the personalized dietary advice. Participants will receive written dietary advice accompanied by a verbal explanation from the nutritionist. Participants can contact the nutritionist at any time by mail or phone in case any question arise. The advice includes the use of regular protein rich food products and protein enriched food products provided by the research team, and will be based on personal dietary preferences. Protein enriched food products that can be incorporated within the regular diet include protein bars, cereals, puddings, coconut water and whey powder, which will be freely provided and shipped to participants' home. Those products can be incorporated in the dietary advice as they can make it easier to increase protein intake due to their high protein content.

For peer review only - http://bmjopen.bmj.com/site/about/guidelines.xhtml

Table 2. Measurements and time of measurements in the PROMISS trial

		Timeline				
		**Screening** (phone)	**Screening visit** (prior baseline)	**Baseline visit**	**3-month FU visit**	**6-month FU visit**
**Topic**	**Specific variables**					
Oral informed consent (phone)		✓				
Screening questionnaire (phone)	Sex, age, self-reported weight, height, eligibility criteria	✓				
Protein intake	Pro55+ screening (38) (phone)	✓				
Protein intake	Combination of three food diaries and three 24-h dietary recalls		✓		✓	✓
Written informed consent			✓			
Cognitive function	MMSE (29)		✓			

For peer review only

Physical functioning	400-m walk test (41, 42)	✓			✓
Antropometrics	Measured body height	✓			
Antropometrics	Measured body weight	✓		✓	✓
Demographic	Education, household		✓		
General characteristics	Perceived health, smoking status		✓		✓
Body composition	Bio-electrical impedance		✓		✓
Body composition	Air displacement plethysmography (Dutch site only)		✓		✓
Physical functioning	SPPB (43)		✓		✓
Muscle strength	Handgrip strength		✓		✓
Muscle strength	Leg extension strength		✓		✓
Self-reported mobility limitations	Ability to walk 400m and climb one flight of stairs		✓	✓	✓
Risk of sarcopenia	SARC-F questionnaire (44)		✓	✓	✓

For peer review only

Malnutrition	BMI < 22.0 kg/m2 or unintentional weight loss > 5% in the last 6 months	✓		✓
Frailty	Frailty Fried Frailty Index (45)	✓		✓
Quality of Life	EuroQol 5D (46)	✓	✓	✓
Health care costs	RUD (47)	✓	✓	✓
Appetite	SNAQ-Appetite (48)	✓		✓
Physical activity	Accelerometers	✓	✓	✓
Process evaluation				✓
Persuasive technology study				
Communication style preferences	Personality traits form	✓		
Usage data of technology	Practical experiences, interaction data of technology (number of notifications, openings, registered food intake, games)		✓	✓
Attitude towards technology	Questionnaire			✓

For peer review only

Microbiota study			
Oral health	Questionnaire	✓	✓
Oral microbiota	Tongue swab (16S rRNA sequencing)	✓	✓
Gut microbiota	Fresh frozen faecal sample (16S rRNA sequencing)	✓	✓
fMRI study			
Oral microbiota	Fasted unstimulated salivary sample (16S rRNA sequencing)	✓	✓
Nutritional and microbial markers	Blood sample	✓	✓
Appetite	VAS-scores of appetite and central neural responses to food-cues measured by fMRI-scan	✓	✓

For peer review only - http://bmjopen.bmj.com/site/about/guidelines.xhtml

Participants receive guidelines how to incorporate the protein enriched food products within their diet. The dietary advice will also incorporate the advice to consume at least one daily meal consisting of ≥ 35 g protein, as studies have shown that this amount increases MPS in older adults (49-51).

Participants in intervention group 2 will receive the same dietary advice as intervention group 1, and the personalized advice to consume at least 7.5-10 g protein through protein (en)rich(ed) food products within half an hour after performing usual physical activity as this may enhance resistance exercise induced MPS (27). One RCT among older adults has shown that protein supplementation in combination with resistance exercise had beneficial effects on e.g. muscle mass and function, but no differences in effect were found between protein consumption pre versus post resistance exercise (52). We therefore recommend protein intake after physical activity as this is a uniform and more feasible advice compared to 'in close proximity of', and might also result in less stomach discomfort as compared to protein consumption prior to physical activity. Usual physical activity is defined as either physical exercise (e.g. biking, swimming, tennis) or the most intensive activities of daily living when the participant does not engage in physical exercise (e.g. gardening, housekeeping, doing groceries) for a minimum of 30 minutes. The advice is linked to most extensive or longest physical activity. Participants are instructed not to become more or less physically active but merely to shift their physical activity or protein intake moment.

During the intervention period, nutritionists will plan follow-up phone calls in consultation with the participants during week 2, week 4, week 8, week 16 and week 20 to ask if they have understood the advice and are able to adhere to the advice. In addition, any issues related to the use of protein enriched food product can be discussed (intervention groups only). If necessary, changes in the dietary advice will be made, for example when weight change > 2 kg has occurred (based on self-assessment). Participants allocated to the

For peer review only

control group do not receive any intervention, but are contacted on similar time points as the intervention groups to ask how they are doing.

All participants are invited to a minimum of one organized lecture on non-health related themes and other social events during the trial in order to stimulate their commitment to the trial. Separate lectures/events (with the same topic) are organized for the intervention groups and the control group to prevent interference between intervention arms. Participants can freely attend those lectures and all travel costs will be reimbursed.

For peer review only

Compliance

We will collect dietary intake prior to the 3-month follow-up visit (by means of the combination of three food diaries and three 24-h dietary recalls) to assess compliance to the dietary advice. This information allows the nutritionists to provide additional advice – if needed – for participants in the intervention groups, which will be provided during the 3-month follow-up visit. Dietary intake will again be assessed at follow-up and compliance to the dietary advice will be determined.

Intervention fidelity

To ensure good adherence to the intervention protocols at both study sites, all personnel working for the trial have undergone extensive training. The nutritionists will follow written standardized operational procedures to develop and provide the personalized dietary advice (with or without additional advice to consume protein within half an hour after usual physical activity). Four times during the conduct of the trial, the nutritionists from one site will visit the other site to attend assessments, and potentially notice and correct differences in order to ensure identical execution of the trial at both sites. In addition, monthly Skype-meetings will be held between all staff involved in the execution of the trial at both sites to solve any

For peer review only - http://bmjopen.bmj.com/site/about/guidelines.xhtml

potential day-to-day issues in a standardized way. Furthermore, identical participant brochures and other printed materials have been developed and translated to Dutch and Finnish language.

Outcomes

Primary outcome

The primary outcome of the PROMISS trial is 6-months change in physical functioning measured by change in walk time using a 400-m walk test (Long Distance Corridor Walk) (41, 42). This test is predictive for higher risk of mortality, incident cardiovascular disease and mobility limitation and disability (53). One advantage of this continuous outcome is that it enables discrimination between categories of risk among participants (54), and it is less prone to a ceiling effect as compared to other functional outcome measures (e.g. SPPB) (55). The course for the 400-m test is 20-m long and marked by a traffic cone and tape line at the beginning and end. For all participants, the test will begin with a mandatory 40-m walk (warm-up) at their usual pace. Thereafter the 400-m test starts with the feet behind and just touching the starting line and ends after 10 complete rounds when one foot is behind the end line. For the 400-m test, older adults will be instructed to walk as fast as possible at a pace they can maintain for 400 m. Standardized encouragement will be given each lap, including the number of laps remaining. At the 6-month follow-up visit, older adults are allowed to use a cane, can take rest as needed (but no rest longer than 60 seconds) and there will be a time limit of 17 minutes. Time will be recorded to the nearest second.

Secondary outcomes

The secondary outcomes are changes in physical performance, muscle strength, body composition, self-reported mobility limitations, QoL, incidence of frailty, incidence of

For peer review only

sarcopenia risk, and incidence of malnutrition. We will also investigate change in health care costs.

Physical performance will be assessed by means of the Short Physical Performance Battery (SPPB) (43). The SPPB assesses lower extremity function which consists of three timed tests: repeated (5x) chair stands test, 4-meter walk test and three standing balance tests (ability to stand with the feet together in the side-by-side, semi-tandem, and tandem positions). The total score ranges from 0-12. A higher score indicates better physical functioning.

Muscle strength will be determined by hand-grip strength (kg). Hand-grip strength is an indicator of overall muscle strength (56) and a higher hand grip strength is associated with decreased risk of physical disabilities (57) and all-cause mortality in old age (57, 58). Maximum grip strength will be measured three times at each hand during baseline and 6-month follow-up visit. We will use a digital dynamometer (Saehan Digital Hand held dynamometer) adjusted for hand size. Participants will be measured in an upright sitting position with the forearms supported by the armrest of a chair according to a standardized protocol (59). The mean of the maximum score of left and right hand will be used for analyses. Muscle strength will also be determined by leg extension strength (N). A higher leg extension strength is associated with decreased risk of mobility disability (60, 61) and lower risk of early mortality (62-64). Leg extension strength will be assessed using a chair designed to measure leg extension strength (65). Maximum leg extension strength will be measured three times for each leg during baseline and 6-month follow-up visit. The mean of the maximum score of left and right leg will be used for analyses.

Body composition will be estimated by means of bioelectrical impedance using the BodyStat 1500MDD devise, using the Kyle equation to determine fat percentage (%), fat mass and fat-free mass (66) and the Sergi equation to determine appendicular skeletal muscle

For peer review only

mass (ASMM, kg) (67). Additionally, at the Dutch study site, body composition (fat free mass (kg), fat mass and fat percentage (%)) will be measured by air displacement plethysmography (68).

Self-reported mobility limitations will be assessed by means of a questionnaire; "Because of your health, how much difficulty do you have walking 400 meter?" and "Because of your health, how much difficulty do you have climbing 10 steps?" Participants will respond using a five level Likert scale: 'no difficulty', 'a little difficulty', 'some difficulty', 'a lot of difficulty' and 'unable to do the activity'. Mobility limitation is defined as two consecutive reports of having any difficulty walking 400 meter or climbing 10 steps without resting due to a health or a physical problem.

QoL will be measured using the EuroQol 5D – 5L questionnaire (46).

Incident frailty will be assessed using the Fried criteria (45). Participants will be considered 'frail' when three or more components are present. Those with no components will be considered 'robust', whereas those with one or two components will be considered 'prefrail'. The criteria include 1) self-reported unintentional weight loss (> 4 kg in past 6 months), 2) self-reported exhaustion (based on two questions from the Center for Epidemiologic Studies Depression (CES-D) scale on exhaustion in the past week at baseline and follow-up: "I felt that everything I did was an effort" and "I could not get going". Scores ranges from 1 'rarely or none of the time' to 4 'always or most of the time'. A score of 3 or 4 on either question indicates exhaustion (69), 3) weakness (grip strength in the lowest 20% of the whole study population based on the mean of the maximum scores, adjusted for gender and BMI), 4) slow walking speed (walk time on the 4m walk test in the slowest 20% of the whole study population, adjusted for gender and height), and 5) low physical activity (total counts per week based on the accelerometer data in the lowest 20% of physical activity for each gender). Incident frailty is considered deterioration of frailty status; i.e. from robust at

For peer review only - http://bmjopen.bmj.com/site/about/guidelines.xhtml

baseline to pre-frail or frail at follow-up or from pre-frail at baseline to frail at follow. Frail participants at baseline will be excluded from these analyses.

Incidence of sarcopenia risk will be assessed with the SARC-F questionnaire (44); how much effort do you experience when 1) lifting and carrying a bag of 4.5 kilo, 2) walking across a room, 3) transferring from a chair or bed, 4) climbing a flight of 10 stairs, and 5) how many times have you fallen in the past year. Answering option include no effort (0 points), a bit of effort (1 point) and a lot of effort (2 points), where a score equal to or greater than 4 is predictive of sarcopenia and poor outcomes. Participants with risk of sarcopenia at baseline will be excluded from these analyses.

Incidence of malnutrition will be defined as BMI < 22.0 kg/m^2 or unintentional weight loss > 5% in the last 6 months. Malnourished participants at baseline will be excluded from these analyses.

A modified version of the Resource Utilization in Dementia Questionnaire (RUD) (47) will be used to collect data on health care and social utilization costs over the period three month prior to baseline, three months prior to the 3-month follow-up visit and three months prior to 6-month follow-up visit. Costs include costs of primary and secondary care, complementary care, informal care and home care.

Other measures

BW will be measured without shoes in underwear to the nearest 0.1 kg using a digital calibrated scale (Finland; SECA 877, the Netherlands; Marsden M-520). Body height will be measured to the nearest millimeter using a SECA stadiometer for mobile height measurements (Finland; SECA 217, the Netherlands; SECA 214). Corrections will be made to adjust the measured body weight for clothing, shoes or a cast (minus 1 kg for each element), and to adjust the measured body height for shoes (minus 1 cm). Change in BW and

For peer review only - http://bmjopen.bmj.com/site/about/guidelines.xhtml

BMI at 3-month and 6-month will be calculated. As the personalized dietary advice is isocaloric, no significant difference in BW or BMI are expected. Physical activity will be objectively assessed by means of an accelerometer (Axivity, AX3) during 7 subsequent days after each clinic visit (baseline, 3-month follow-up visit and 6-month follow-up visit). The accelerometer will be attached by a nutritionist to the frontal part of the right thigh in the mid-point between iliac crest and patella bone when sitting down, with a surgical plaster. Participants can perform any physical activity as the accelerometer is water resistant. Appetite will be measured with SNAQ-Appetite questionnaire (48). Dietary intake will be assessed by means of a combination of three food diaries and three 24-h dietary recalls prior the 3-month follow-up visit and the 6-month follow-up visit.

Sample size and statistical analyses

<u>Sample size</u>

The study is powered to detect a substantial meaningful change of 28 sec (SD=61 sec) (70) between the respective intervention groups and the control group on the primary outcome walk time on the 400-m walk test, assuming a 2-sided test at α=0.05 with a power of 0.8. For this, 75 participants per group are needed, which is 225 in total. Assuming a drop-out of 15% (which was reported in a comparable study of Bhasin et al. 2018 (19), the total number of study participants to be included in the study is n=264. Therefore, we aim to include a total of n=132 at each study site (n=44 per study group per study site).

<u>Statistical analyses plan</u>

Statistical reporting will be according to the CONSORT standards (71). The collected data at the two study sites will be pooled together at the Amsterdam site, with a variable indicating study site. All statistical analyses on primary and secondary outcome measures will be

For peer review only

502 performed by an independent statistician blinded for group allocation. Baseline
503 characteristics will be described (percentages, means ± standard deviations) by study group.
504 The primary analyses will be based on the intention-to-treat principle, i.e. data from
505 participants allocated to the intervention groups will be analyzed as part of those groups,
506 irrespectively of their level of adherence to the advice. Multiple Imputation using
507 multivariate Imputation by Chained Equations will be used to impute missing cost and effect
508 data. The continuous primary outcome (change in 400-m walk time) will be analyzed using
509 mixed model regression analyses with study site as random variable. We will adjust for
510 baseline 400-m walk time as well as baseline protein intake (g/kg aBW/d) and sex (the
511 stratification factors for randomization). We will compare intervention effects of the
512 respective intervention groups versus the control group. Effects will also be expressed in
513 Cohen's d and the corresponding 95% confidence intervals will be calculated, which allows
514 comparison between intervention effect estimates between different outcome measures. The
515 secondary outcomes and other measures will be analyzed using mixed model regression
516 analogously to the primary outcome. For binary secondary outcomes, generalized estimating
517 equation models will be used. With regard to time-to-event analyses (incident sarcopenia risk
518 and mobility limitations) Cox proportional hazard models will be used. Time-to-event is
519 defined as the time of the start of the study period to the date of the first occurrence of the
520 event (3-month follow-up visit or 6-month follow-up visit). Participants who do not meet
521 these criteria will be censored at the latest time we had information available. We will
522 perform subgroup analyses stratified by baseline protein intake (< 0.9 or 0.9-1.0 g/kg
523 aBW/d), sex and baseline 400-m time (based on median) for the primary and secondary
524 outcomes.
525 Per-protocol analyses will also be conducted as a sensitivity analysis. Effect estimates
526 for change in primary and secondary outcome measures will be calculated for participants

For peer review only - http://bmjopen.bmj.com/site/about/guidelines.xhtml

527 from the intervention groups who reached the protein target of at least 1.2 g/kg aBW/d after
528 both 3 and after 6 months (mean protein intake based on the three 24-h dietary recalls) vs.
529 participants from the control group. Data will be analyzed using SPSS (IBM SPSS Statistics.
530 Armonk, NY). A two-sided *P*-value of 0.05 is considered statistical significant.
531 The cost-effectiveness analysis will be performed from a healthcare perspective. Total
532 mean costs during the study will be related to physical functioning (the 400-m walk test) and
533 change in Quality-Adjusted Life-Years based on the EuroQol 5D questionnaire. Mixed model
534 regression analyses will be used to estimate differences in the primary outcome of the
535 respective interventions groups versus the control group. Linear regression analyses will be
536 used to estimate differences in QoL (expressed as Quality-Adjusted Life-Years) and
537 healthcare costs. Incremental cost-effectiveness ratios will be calculated by dividing the
538 difference in costs by the difference in effects. Statistical uncertainty will be estimated using
539 bias-corrected accelerated bootstrapping (5000 replications) and will be presented using cost-
540 effectiveness planes and cost-effectiveness acceptability curves.
541
542 *Participants' safety*
543 In case any (medical) questions arise during the screening or intervention period, participants
544 can consult an independent medical doctor. All adverse events and serious adverse advents
545 will be tracked by the nutritionists during the follow-up phone calls, 3-month follow-up visit
546 and 6-month follow-up visit to assess their potential relationship to the intervention at both
547 sites and will documented in the final report. Adverse events will be reported within 7 days
548 (death or life threatening situations) or within 15 days (in case of other adverse events) of
549 first knowledge to The Medical Ethical Committee of the Amsterdam UMC, location Vumc
550 (required for the Dutch site only).
551

For peer review only

Data quality assurance and data management

Research data will be collected at each site and each visit (baseline, 3-month follow-up visit and 6-month follow-up visit) using a standardized protocol with the same order of assessments, and entered twice in separate electronic datasets. When discrepancies between the datasets are found, the original questionnaire will be consulted. Questionnaire items and measurements will include the corresponding variable names to minimize errors in data entering. Finally the two final electronic dataset (one from each site) containing all data will be pooled. A data catalogue and codebook will be developed.

Original questionnaires will be stored in a secure manner at each site in an area with limited access. All records that contain names (i.e. informed consent forms), will be stored separately from study records identified by code number. All databases will be secured with password-protected access systems.

Patient and Public Involvement

The PROMISS randomized controlled trial is designed by the Faculty of Science (VU Amsterdam, the Netherlands) and the Department of General Practice and Primary Health Care (University of Helsinki, Finland) (please see Appendix I for the PROMIS trial group), a collaboration of the EU Horizon 2020 PROMISS Project. Two medical and one ethical advisor are involved in the study. As part of the PROMISS project, we previously performed three pilot studies as a preparation of the long term PROMISS randomized trial, of which one is published (72). We included the feedback of the participants in designing the long term PROMISS randomized trial; participants enjoyed participating in the pilot studies and they liked the frequent contact with the nutritionist. We also tested which protein enriched food products they preferred and included those products in the long term PROMISS randomized trial which they liked the most. Older adults are not involved in recruitment of participants or

For peer review only - http://bmjopen.bmj.com/site/about/guidelines.xhtml

conduct of the study. Results of this study will be disseminated to participants through sending them a lay abstract with the results and conclusions of the study. At the end of the study, each participant will receive a fact-sheet with personal results of dietary intake data, hand grip strenght, body composition mesures and body weight. Participant burden of the pilot intervention was assessed using informal feedback from older adults participating in one of three pilot studies.

Ancillary studies

Within the PROMISS trial, three ancillary studies will be conducted: 1) persuasive technology study, 2) microbiota study, and 3) fMRI study.

1. Persuasive technology study

The primary aim of this study is to examine the effect of persuasive technology on adherence to the personalized dietary advice aiming at increasing protein intake to at least 1.2 g/kg aBW/d in a sub-sample of Dutch participants from intervention group 1 (n=24) and intervention group 2 (n=24), i.e. the first 24 participants of intervention group 1 and the first 24 participants of intervention group 2 that consent to it (writting informed consent will be signed).

Participants will be provided with a food storage box that registers which provided protein enriched food products are taken out. The food box is used to store the protein enriched food products provided by the research team. Participants will also receive a tablet that allows participants to register any consumed protein enriched food products and supports them in finding alternative food products that contain a comparable amount of protein. For this, the system uses the personalized dietary advice as provided by the nutritionist and data from the storage box. The tablet application aims to stimulate adherence to the dietary advice

For peer review only

For peer review only - http://bmjopen.bmj.com/site/about/guidelines.xhtml

by providing tailored and personalized messages. In addition, personality characteristics and communication style preferences that are determined via a questionnaire completed at baseline are used to tailor the style and tone of these messages (73).

In addition to the personalized messages, half of the participants from intervention group 1 and 2 who participate in the persuasive technology sub-study will also receive a gamified version of the tablet application (n=12 + n=12). In this version, participants can earn game-points by registering their consumed protein (en)rich(ed) food products and by playing mini-games about the protein content of foods (i.e. guess the protein content, more-or-less protein). The distribution of receiving the gamified version vs. standard version is quasi-randomized, where we will balance the group size.

At the consultation meeting, participants receive their food storage box and tablet. Both are fully configured, i.e. they are loaded with their personal dietary advice. After the 6-month follow-up visit, participants will be asked to return the equipment and fill out questions on the feasibility and user experience of the provided persuasive technology.

The secondary objectives are 1) to investigate to what extent participants perceive messages of which the style and tone are adapted to their personal characteristics as personalized and adequate, and 2) to determine the effect of gamification on the effectiveness and feasibility of the persuasive technology.

<u>2. Microbiota study</u>

In the microbiota study, the effect of personalized dietary advice aiming at increasing protein intake in community-dwelling older adults with lower habitual protein intake on both the oral and gut microbiota is investigated. The study will be conducted at both study sites.

The human microbiota consists of the $4*10^{13}$ micro-organisms that inhabit the body (74). The emergence of next generation DNA sequencing techniques at the start of the 21st

For peer review only

century has allowed more detailed study of the microbiota and since then, the microbiota composition has been associated with both health and disease (75), as well as aging itself (76, 77). Moreover, several interventional studies proved that dietary changes also affect the gut microbiota, with the first microbial shifts being evident within 48 hours (78). The altered microbiota in turn, can differentially affect the human host metabolism through the production of metabolically active metabolites. Less is known about the oral microbiota. It was found to be associated with oral health and function and even nutritional status (79, 80), but its possible role in undernutrition in older adults has not been investigated.

A fresh frozen fecal sample and tongue swab is collected at baseline and 6-month follow-up visit once written informed consent is provided. Participants from either the control group or intervention group 1 can be included in this study. Participants from the intervention group 2 are excluded to limit the number of groups and parameters in this exploratory study. Additional exclusion criteria are: use of systemic antibiotics in the three months prior to the first sampling visit, diagnosis with inflammatory bowel disease and prolonged institutionalization (> 4 weeks) in the three months prior to the first sampling visit. There is no restriction other than consent rate to the number of PROMISS participants that will be included in this side study.

Once all samples from all participants are collected, fecal samples are shipped to the Wallenberg Laboratory of Cardiovascular and Metabolic research (at the University of Gothenburg, in Sweden) for 16S rRNA sequencing using sequencing methods previously described (81). The tongue swabs will be send to the Netherlands Organisation for Applied Scientific Research for 16S rRNA sequencing as is previously described (82).

<u>3. fMRI study</u>

For peer review only - http://bmjopen.bmj.com/site/about/guidelines.xhtml

In the fMRI study, we will investigate the effect of personalized dietary advice aiming at increasing protein intake in community-dwelling older adults with lower habitual protein intake on central brain circuits involved in the regulation of appetite. Several studies demonstrated that increasing protein intake affects appetite (83) and the gut microbiota (84). However, none have studied the effects on both simultaneously, or the interaction. A functional MRI (fMRI) scan will be used to measure the brain responses to visual or actual food cues. Brain activity in response to food cues will also be related to (shifts) in the gut microbiota. Therefore, only participants from the microbiota side study can be included in this study, with additional exclusion criteria: being claustrophobic, being diagnosed with a mental disorder (e.g. depression or addiction), being uncorrectable visually or hearing impaired, or having a contra-indication for MRI-scans (e.g. having a pacemaker). Up to 50 participants will be included in this side study. This side study will only be conducted at the Dutch study site.

Once written informed consent is provided, participants who are included in this side study will be asked to visit the Amsterdam University Medical Centre, location VUmc, for an fMRI scan twice during the study period: at baseline and at 6-month follow-up visit. Prior to the fMRI-scan, additional salivary and blood samples will be collected for determination of additional nutritional and microbial biomarkers. The protocol for the fMRI experiments have been previously described (85, 86).

Discussion

There is an ongoing discussion whether the EFSA RDA of 0.8 g protein /kg BW/d is sufficient for older adults and whether it should be increased to at least 1.0-1.2 g protein/kg BW/d to support muscle health and functioning. National guidelines of some European countries already increased their RDA, i.e. the RDA of the German-speaking countries (D-A-

For peer review only

For peer review only - http://bmjopen.bmj.com/site/about/guidelines.xhtml

CH) is increased to 1.0 g/kg BW/d (87), and the Nordic Nutrition Recommendation has increased their RDA to 1.2 g/kg BW/d (88). The PROMISS trial is the first RCT which will investigate the effect of personalized dietary advice aiming at increasing protein intake and the combined effect of personalized dietary advice aiming at increasing protein and the timing of protein intake in close proximity of usual physical activity, on change in physical functioning after 6 months among community-dwelling older adults (≥ 65 y) with a habitual protein intake of < 1.0 g/kg adjusted (a)BW/d. The PROMISS trial will therefore provide additional insight to the question whether the current EFSA RDA for protein for older adults should be increased to 1.2 g/kg aBW/d, and whether optimal timing of protein intake will additionally benefit physical functioning.

A strong and unique aspect of the PROMISS trial is that we will include participants with a habitual protein intake < 1.0 g/kg aBW/d, excluding those with a BMI < 18.5 and > 32.0 kg/m^2. This will allow us to examine the effects of increasing protein intake from < 1.0 to at least 1.2 g/kg aBW/d. An innovative component of our study is that we will investigate the combined benefit of increasing protein intake and timing of protein intake with usual physical activity on physical functioning and other health related outcomes. Another strength is that in our study the intervention is based on personalized dietary advice which is likely more feasible in the long term to maintain in everyday life, compared to providing custom-prepared meals (19) or protein supplements (89, 90), as done in most other studies. Finally, we will be able to investigate the effect of persuasive technology on adherence to the dietary advice strategy, and the effect of the dietary advice on the microbiota composition and on central responses to food-cues in brain areas involved in appetite regulation. One limitation of this study is that the biological value of the total protein intake (i.e. type of amino acids) is unknown. Another limitation is that the duration of the trial might not be long enough to

For peer review only

For peer review only - http://bmjopen.bmj.com/site/about/guidelines.xhtml

observe a sufficient amount of incident cases of e.g. risk of malnutrition, frailty or risk of sarcopenia.

In summary, this randomized controlled trial will demonstrate the effectiveness of personalized dietary advice aiming at increasing protein intake to at least 1.2 g protein/kg BW/d on physical functioning in older adults with a lower habitual protein intake, with or without the advice to consume protein in close proximity of usual physical activity.

Ethics and disseminations

The study has been approved by the Ethics Committee of the Helsinki University Central Hospital, Finland (ID of the approval; HUS/1530/2018) and The Medical Ethical Committee of the Amsterdam UMC, location VUmc, Amsterdam, the Netherlands (ID of the approval; 2018.399). Oral informed consent will be obtained from each participants before the screening procedure and written informed (please see appendix II) consent will be obtained from each participant before any measurement take place. Personal data were not identifiable during the analysis.

Results will be send to national and international conferences and will submitted for publication in peer-reviewed journals. In addition, lay abstracts will be made available for participants and the public. Links to research output and dissemination activities will be made available on the PROMISS website, available at www.promiss-vu.eu and social media channels.

For peer review only

For peer review only - http://bmjopen.bmj.com/site/about/guidelines.xhtml

References

1. EFSA. Scientific Opinion on Dietary Reference Values for protein. EFSA Panel on Dietetic Products, Nutrition and Allergies (NDA). EFSA Journal. 2012;10:2257.
2. Bauer J, Biolo G, Cederholm T, Cesari M, Cruz-Jentoft AJ, Morley JE, et al. Evidence-based recommendations for optimal dietary protein intake in older people: a position paper from the PROT-AGE Study Group. J Am Med Dir Assoc. 2013;14:542-559.
3. Deutz NE, Bauer JM, Barazzoni R, Biolo G, Boirie Y, Bosy-Westphal A, et al. Protein intake and exercise for optimal muscle function with aging: recommendations from the ESPEN Expert Group. Clin Nutr. 2014;33:929-936.
4. Cuthbertson D, Smith K, Babraj J, Leese G, Waddell T, Atherton P, et al. Anabolic signaling deficits underlie amino acid resistance of wasting, aging muscle. FASEB J. 2005;19:422-424.
5. Moore DR, Churchward-Venne TA, Witard O, Breen L, Burd NA, Tipton KD, et al. Protein ingestion to stimulate myofibrillar protein synthesis requires greater relative protein intakes in healthy older versus younger men. J Gerontol A Biol Sci Med Sci. 2015;70:57-62.
6. Wall BT, Gorissen SH, Pennings B, Koopman R, Groen BB, Verdijk LB, et al. Aging Is Accompanied by a Blunted Muscle Protein Synthetic Response to Protein Ingestion. PLoS One. 2015;10:e0140903.
7. Murphy CH, Saddler NI, Devries MC, McGlory C, Baker SK, Phillips SM. Leucine supplementation enhances integrative myofibrillar protein synthesis in free-living older men consuming lower- and higher-protein diets: a parallel-group crossover study. Am J Clin Nutr. 2016;104:1594-1606.

For peer review only

8. Holwerda AM, Paulussen KJM, Overkamp M, Goessens JPB, Kramer IF, Wodzig W, et al. Dose-Dependent Increases in Whole-Body Net Protein Balance and Dietary Protein-Derived Amino Acid Incorporation into Myofibrillar Protein During Recovery from Resistance Exercise in Older Men. J Nutr. 2019;149:221-230.

9. Houston DK, Nicklas BJ, Ding J, Harris TB, Tylavsky FA, Newman AB, et al. Dietary protein intake is associated with lean mass change in older, community-dwelling adults: the Health, Aging, and Body Composition (Health ABC) Study. Am J Clin Nutr. 2008;87:150-155.

10. Houston DK, Tooze JA, Garcia K, Visser M, Rubin S, Harris TB, et al. Protein Intake and Mobility Limitation in Community-Dwelling Older Adults: the Health ABC Study. J Am Geriatr Soc. 2017;65:1705-1711.

11. Gray-Donald K, St-Arnaud-McKenzie D, Gaudreau P, Morais JA, Shatenstein B, Payette H. Protein intake protects against weight loss in healthy community-dwelling older adults. J Nutr. 2014;144:321-326.

12. Mendonca N, Granic A, Hill TR, Siervo M, Mathers JC, Kingston A, et al. Protein Intake and Disability Trajectories in Very Old Adults: The Newcastle 85+ Study. J Am Geriatr Soc. 2019;67:50-56.

13. Ten Haaf DSM, Nuijten MAH, Maessen MFH, Horstman AMH, Eijsvogels TMH, Hopman MTE. Effects of protein supplementation on lean body mass, muscle strength, and physical performance in nonfrail community-dwelling older adults: a systematic review and meta-analysis. Am J Clin Nutr. 2018;108:1043-1059.

14. Layman DK. Dietary Guidelines should reflect new understandings about adult protein needs. Nutr Metab (Lond). 2009;6:12.

15. Paddon-Jones D, Campbell WW, Jacques PF, Kritchevsky SB, Moore LL, Rodriguez NR, et al. Protein and healthy aging. Am J Clin Nutr. 2015;

For peer review only

16. Park Y, Choi JE, Hwang HS. Protein supplementation improves muscle mass and physical performance in undernourished prefrail and frail elderly subjects: a randomized, double-blind, placebo-controlled trial. Am J Clin Nutr. 2018;108:1026-1033.

17. Ten Haaf DSM, Eijsvogels TMH, Bongers C, Horstman AMH, Timmers S, de Groot L, et al. Protein supplementation improves lean body mass in physically active older adults: a randomized placebo-controlled trial. J Cachexia Sarcopenia Muscle. 2019;

18. Beelen J, de Roos NM, de Groot L. A 12-week intervention with protein-enriched foods and drinks improved protein intake but not physical performance of older patients during the first 6 months after hospital release: a randomised controlled trial. Br J Nutr. 2017;117:1541-1549.

19. Bhasin S, Apovian CM, Travison TG, Pencina K, Moore LL, Huang G, et al. Effect of Protein Intake on Lean Body Mass in Functionally Limited Older Men: A Randomized Clinical Trial. JAMA Intern Med. 2018;178:530-541.

20. Cermak NM, Res PT, de Groot LC, Saris WH, van Loon LJ. Protein supplementation augments the adaptive response of skeletal muscle to resistance-type exercise training: a meta-analysis. Am J Clin Nutr. 2012;96:1454-1464.

21. Finger D, Goltz FR, Umpierre D, Meyer E, Rosa LH, Schneider CD. Effects of protein supplementation in older adults undergoing resistance training: a systematic review and meta-analysis. Sports Med. 2015;45:245-255.

22. Rondanelli M, Klersy C, Terracol G, Talluri J, Maugeri R, Guido D, et al. Whey protein, amino acids, and vitamin D supplementation with physical activity increases fat-free mass and strength, functionality, and quality of life and decreases inflammation in sarcopenic elderly. Am J Clin Nutr. 2016;103:830-840.

For peer review only

23. Liao CD, Tsauo JY, Wu YT, Cheng CP, Chen HC, Huang YC, et al. Effects of protein supplementation combined with resistance exercise on body composition and physical function in older adults: a systematic review and meta-analysis. Am J Clin Nutr. 2017;106:1078-1091.

24. Englund DA, Kirn DR, Koochek A, Zhu H, Travison TG, Reid KF, et al. Nutritional Supplementation With Physical Activity Improves Muscle Composition in Mobility-Limited Older Adults, The VIVE2 Study: A Randomized, Double-Blind, Placebo-Controlled Trial. J Gerontol A Biol Sci Med Sci. 2017;73:95-101.

25. Mori H, Tokuda Y. Effect of whey protein supplementation after resistance exercise on the muscle mass and physical function of healthy older women: A randomized controlled trial. Geriatr Gerontol Int. 2018;18:1398-1404.

26. Seino S, Sumi K, Narita M, Yokoyama Y, Ashida K, Kitamura A, et al. Effects of Low-Dose Dairy Protein Plus Micronutrient Supplementation during Resistance Exercise on Muscle Mass and Physical Performance in Older Adults: A Randomized, Controlled Trial. J Nutr Health Aging. 2018;22:59-67.

27. Cribb PJ, Hayes A. Effects of supplement timing and resistance exercise on skeletal muscle hypertrophy. Med Sci Sports Exerc. 2006;38:1918-1925.

28. Saunders JB, Aasland OG, Babor TF, de la Fuente JR, Grant M. Development of the Alcohol Use Disorders Identification Test (AUDIT): WHO Collaborative Project on Early Detection of Persons with Harmful Alcohol Consumption--II. Addiction. 1993;88:791-804.

29. Folstein MF, Folstein SE, McHugh PR. "Mini-mental state". A practical method for grading the cognitive state of patients for the clinician. J Psychiatr Res. 1975;12:189-198.

For peer review only

For peer review only - http://bmjopen.bmj.com/site/about/guidelines.xhtml

30. Cederholm T, Jensen GL, Correia M, Gonzalez MC, Fukushima R, Higashiguchi T, et al. GLIM criteria for the diagnosis of malnutrition - A consensus report from the global clinical nutrition community. Clin Nutr. 2019;38:1-9.

31. Kim S, Leng XI, Kritchevsky SB. Body Composition and Physical Function in Older Adults with Various Comorbidities. Innov Aging. 2017;1:igx008.

32. Vincent HK, Vincent KR, Lamb KM. Obesity and mobility disability in the older adult. Obes Rev. 2010;11:568-579.

33. Kritchevsky SB, Beavers KM, Miller ME, Shea MK, Houston DK, Kitzman DW, et al. Intentional weight loss and all-cause mortality: a meta-analysis of randomized clinical trials. PLoS One. 2015;10:e0121993.

34. Fouque D, Aparicio M. Eleven reasons to control the protein intake of patients with chronic kidney disease. Nat Clin Pract Nephrol. 2007;3:383-392.

35. Fouque D, Pelletier S, Mafra D, Chauveau P. Nutrition and chronic kidney disease. Kidney Int. 2011;80:348-357.

36. Eyre S, Attman PO, Haraldsson B. Positive effects of protein restriction in patients with chronic kidney disease. J Ren Nutr. 2008;18:269-280.

37. Berner LA, Becker G, Wise M, Doi J. Characterization of dietary protein among older adults in the United States: amount, animal sources, and meal patterns. J Acad Nutr Diet. 2013;113:809-815.

38. Wijnhoven HAH, Elstgeest LEM, de Vet HCW, Nicolaou M, Snijder MB, Visser M. Development and validation of a short food questionnaire to screen for low protein intake in community-dwelling older adults: The Protein Screener 55+ (Pro55+). PLoS One. 2018;13:e0196406.

39. The National Institute for Health and Welfare. Fineli: the National Food Composition Database in Finland. [Available from: https://fineli.fi/fineli/en/index?

For peer review only

40. Nederlands Voedingsstoffenbestand 2016 (Dutch Food Composition Table 2016): Voedingscentrum 2016; [Available from: https://nevo-online.rivm.nl/.

41. Simonsick EM, Montgomery PS, Newman AB, Bauer DC, Harris T. Measuring fitness in healthy older adults: the Health ABC Long Distance Corridor Walk. J Am Geriatr Soc. 2001;49:1544-1548.

42. Simonsick EM, Fan E, Fleg JL. Estimating cardiorespiratory fitness in well-functioning older adults: treadmill validation of the long distance corridor walk. J Am Geriatr Soc. 2006;54:127-132.

43. Guralnik JM, Simonsick EM, Ferrucci L, Glynn RJ, Berkman LF, Blazer DG, et al. A short physical performance battery assessing lower extremity function: association with self-reported disability and prediction of mortality and nursing home admission. J Gerontol. 1994;49:M85-94.

44. Malmstrom TK, Morley JE. SARC-F: a simple questionnaire to rapidly diagnose sarcopenia. J Am Med Dir Assoc. 2013;14:531-532.

45. Fried LP, Tangen CM, Walston J, Newman AB, Hirsch C, Gottdiener J, et al. Frailty in older adults: evidence for a phenotype. J Gerontol A Biol Sci Med Sci. 2001;56:M146-156.

46. EuroQol G. EuroQol--a new facility for the measurement of health-related quality of life. Health Policy. 1990;16:199-208.

47. Wimo A. Evaluation of the resource utilization and caregiver time in Anti-dementia drug trials-a quantitative battery. The health economics of dementia. 1998;

48. Wilson MM, Thomas DR, Rubenstein LZ, Chibnall JT, Anderson S, Baxi A, et al. Appetite assessment: simple appetite questionnaire predicts weight loss in community-dwelling adults and nursing home residents. Am J Clin Nutr. 2005;82:1074-1081.

For peer review only

For peer review only - http://bmjopen.bmj.com/site/about/guidelines.xhtml

49. English KL, Paddon-Jones D. Protecting muscle mass and function in older adults during bed rest. Curr Opin Clin Nutr Metab Care. 2010;13:34-39.

50. Paddon-Jones D, Rasmussen BB. Dietary protein recommendations and the prevention of sarcopenia. Curr Opin Clin Nutr Metab Care. 2009;12:86-90.

51. Deutz NE, Wolfe RR. Is there a maximal anabolic response to protein intake with a meal? Clin Nutr. 2013;32:309-313.

52. Nabuco HCG, Tomeleri CM, Sugihara Junior P, Fernandes RR, Cavalcante EF, Antunes M, et al. Effects of Whey Protein Supplementation Pre- or Post-Resistance Training on Muscle Mass, Muscular Strength, and Functional Capacity in Pre-Conditioned Older Women: A Randomized Clinical Trial. Nutrients. 2018;10:

53. Newman AB, Simonsick EM, Naydeck BL, Boudreau RM, Kritchevsky SB, Nevitt MC, et al. Association of long-distance corridor walk performance with mortality, cardiovascular disease, mobility limitation, and disability. JAMA. 2006;295:2018-2026.

54. Beaudart C, Rolland Y, Cruz-Jentoft AJ, Bauer JM, Sieber C, Cooper C, et al. Assessment of Muscle Function and Physical Performance in Daily Clinical Practice : A position paper endorsed by the European Society for Clinical and Economic Aspects of Osteoporosis, Osteoarthritis and Musculoskeletal Diseases (ESCEO). Calcif Tissue Int. 2019;105:1-14.

55. Sayers SP, Guralnik JM, Newman AB, Brach JS, Fielding RA. Concordance and discordance between two measures of lower extremity function: 400 meter self-paced walk and SPPB. Aging Clin Exp Res. 2006;18:100-106.

56. Rantanen T, Era P, Heikkinen E. Maximal Isometric Muscle Strength and Socioeconomic Status, Health, and Physical Activity in 75-Year-Old Persons. Journal of Aging and Physical Activity. 1994;2:206-220.

For peer review only

For peer review only - http://bmjopen.bmj.com/site/about/guidelines.xhtml

57. Rijk JM, Roos PR, Deckx L, van den Akker M, Buntinx F. Prognostic value of handgrip strength in people aged 60 years and older: A systematic review and meta-analysis. Geriatr Gerontol Int. 2016;16:5-20.

58. Wu Y, Wang W, Liu T, Zhang D. Association of Grip Strength With Risk of All-Cause Mortality, Cardiovascular Diseases, and Cancer in Community-Dwelling Populations: A Meta-analysis of Prospective Cohort Studies. J Am Med Dir Assoc. 2017;18:551 e517-551 e535.

59. Roberts HC, Denison HJ, Martin HJ, Patel HP, Syddall H, Cooper C, et al. A review of the measurement of grip strength in clinical and epidemiological studies: towards a standardised approach. Age Ageing. 2011;40:423-429.

60. Visser M, Goodpaster BH, Kritchevsky SB, Newman AB, Nevitt M, Rubin SM, et al. Muscle mass, muscle strength, and muscle fat infiltration as predictors of incident mobility limitations in well-functioning older persons. J Gerontol A Biol Sci Med Sci. 2005;60:324-333.

61. Reinders I, Murphy RA, Koster A, Brouwer IA, Visser M, Garcia ME, et al. Muscle Quality and Muscle Fat Infiltration in Relation to Incident Mobility Disability and Gait Speed Decline: the Age, Gene/Environment Susceptibility-Reykjavik Study. J Gerontol A Biol Sci Med Sci. 2015;70:1030-1036.

62. Reinders I, Murphy RA, Brouwer IA, Visser M, Launer L, Siggeirsdottir K, et al. Muscle Quality and Myosteatosis: Novel Associations With Mortality Risk: The Age, Gene/Environment Susceptibility (AGES)-Reykjavik Study. Am J Epidemiol. 2016;183:53-60.

63. Volaklis KA, Halle M, Meisinger C. Muscular strength as a strong predictor of mortality: A narrative review. Eur J Intern Med. 2015;26:303-310.

For peer review only

For peer review only - http://bmjopen.bmj.com/site/about/guidelines.xhtml

64. Garcia-Hermoso A, Cavero-Redondo I, Ramirez-Velez R, Ruiz JR, Ortega FB, Lee DC, et al. Muscular Strength as a Predictor of All-Cause Mortality in an Apparently Healthy Population: A Systematic Review and Meta-Analysis of Data From Approximately 2 Million Men and Women. Arch Phys Med Rehabil. 2018;99:2100-2113 e2105.

65. Gerrits HL, Hopman MT, Sargeant AJ, de Haan A. Reproducibility of contractile properties of the human paralysed and non-paralysed quadriceps muscle. Clin Physiol. 2001;21:105-113.

66. Kyle UG, Genton L, Karsegard L, Slosman DO, Pichard C. Single prediction equation for bioelectrical impedance analysis in adults aged 20--94 years. Nutrition. 2001;17:248-253.

67. Sergi G, De Rui M, Veronese N, Bolzetta F, Berton L, Carraro S, et al. Assessing appendicular skeletal muscle mass with bioelectrical impedance analysis in free-living Caucasian older adults. Clin Nutr. 2015;34:667-673.

68. McCrory MA, Gomez TD, Bernauer EM, Mole PA. Evaluation of a new air displacement plethysmograph for measuring human body composition. Med Sci Sports Exerc. 1995;27:1686-1691.

69. Radloff LS. The CES-D Scale: A Self-Report Depression Scale for Research in the General Population. Appl Psychol Meas. 1977;1:385-401.

70. Perera S, Studenski S, Newman A, Simonsick E, Harris T, Schwartz A, et al. Are estimates of meaningful decline in mobility performance consistent among clinically important subgroups? (Health ABC study). J Gerontol A Biol Sci Med Sci. 2014;69:1260-1268.

71. Schulz KF, Altman DG, Moher D, Group C. CONSORT 2010 Statement: updated guidelines for reporting parallel group randomised trials. BMC Med. 2010;8:18.

For peer review only

For peer review only - http://bmjopen.bmj.com/site/about/guidelines.xhtml

72. Reinders I, Visser M, Wijnhoven HAH. Two dietary advice strategies to increase protein intake among community-dwelling older adults: A feasibility study. Clin Nutr ESPEN. 2020;37:157-167.

73. Lubbe LMvd, Klein MCA. Designing a system with persuasive communication to improve diet compliance for elderly users. Proceedings of the 13th EAI International Conference on Pervasive Computing Technologies for Healthcare; Trento, Italy. 3329217: ACM; 2019. p. 234-241.

74. Sender R, Fuchs S, Milo R. Are We Really Vastly Outnumbered? Revisiting the Ratio of Bacterial to Host Cells in Humans. Cell. 2016;164:337-340.

75. Schroeder BO, Backhed F. Signals from the gut microbiota to distant organs in physiology and disease. Nat Med. 2016;22:1079-1089.

76. Biagi E, Nylund L, Candela M, Ostan R, Bucci L, Pini E, et al. Through ageing, and beyond: gut microbiota and inflammatory status in seniors and centenarians. PLoS One. 2010;5:e10667.

77. Odamaki T, Kato K, Sugahara H, Hashikura N, Takahashi S, Xiao JZ, et al. Age-related changes in gut microbiota composition from newborn to centenarian: a cross-sectional study. BMC Microbiol. 2016;16:90.

78. David LA, Maurice CF, Carmody RN, Gootenberg DB, Button JE, Wolfe BE, et al. Diet rapidly and reproducibly alters the human gut microbiome. Nature. 2014;505:559-563.

79. Yamashita Y, Takeshita T. The oral microbiome and human health. J Oral Sci. 2017;59:201-206.

80. Testa M, Erbiti S, Delgado A, Cardenas IL. Evaluation of oral microbiota in undernourished and eutrophic children using checkerboard DNA-DNA hybridization. Anaerobe. 2016;42:55-59.

For peer review only

For peer review only - http://bmjopen.bmj.com/site/about/guidelines.xhtml

970 81. Deschasaux M, Bouter KE, Prodan A, Levin E, Groen AK, Herrema H, et al. Depicting the composition of gut microbiota in a population with varied ethnic origins but shared geography. Nat Med. 2018;24:1526-1531.

973 82. Bosch A, Levin E, van Houten MA, Hasrat R, Kalkman G, Biesbroek G, et al. Development of Upper Respiratory Tract Microbiota in Infancy is Affected by Mode of Delivery. EBioMedicine. 2016;9:336-345.

976 83. Mollahosseini M, Shab-Bidar S, Rahimi MH, Djafarian K. Effect of whey protein supplementation on long and short term appetite: A meta-analysis of randomized controlled trials. Clinical nutrition ESPEN. 2017;20:34-40.

979 84. van de Wouw M, Schellekens H, Dinan TG, Cryan JF. Microbiota-Gut-Brain Axis: Modulator of Host Metabolism and Appetite. J Nutr. 2017;147:727-745.

981 85. van Bloemendaal L, RG IJ, Ten Kulve JS, Barkhof F, Konrad RJ, Drent ML, et al. GLP-1 receptor activation modulates appetite- and reward-related brain areas in humans. Diabetes. 2014;63:4186-4196.

984 86. van Bloemendaal L, Veltman DJ, Ten Kulve JS, Groot PF, Ruhe HG, Barkhof F, et al. Brain reward-system activation in response to anticipation and consumption of palatable food is altered by glucagon-like peptide-1 receptor activation in humans. Diabetes Obes Metab. 2015;17:878-886.

988 87. Nutrition reference intake values for the German Speaking Countries D-A-CH ed 2, update 1 Bonn2016 [Available from: https://www.dge.de/wissenschaft/referenzwerte/protein/.

991 88. Nordic Council of Ministers. Nordic nutrition recommendations 2012. Integrating nutrition physical activity; 2014. pp. 1e627. Copenhagen.

993 89. Tieland M, Franssen R, Dullemeijer C, van Dronkelaar C, Kyung Kim H, Ispoglou T, et al. The Impact of Dietary Protein or Amino Acid Supplementation on Muscle Mass

For peer review only

and Strength in Elderly People: Individual Participant Data and Meta-Analysis of RCT's. J Nutr Health Aging. 2017;21:994-1001.

90. Reinders I, Volkert D, de Groot L, Beck AM, Feldblum I, Jobse I, et al. Effectiveness of nutritional interventions in older adults at risk of malnutrition across different health care settings: Pooled analyses of individual participant data from nine randomized controlled trials. Clin Nutr. 2019;38:1797-1806.

For peer review only

For peer review only - http://bmjopen.bmj.com/site/about/guidelines.xhtml

Author Contributions

IR, *HAHW*, *IAB*, *MRO* and *MV* obtained funding for the PROMISS project. *IR* and *HAHW* coordinate the trial center at the Vrije Universiteit Amsterdam, the Netherlands. *SKJ* and *MHS* coordinate the trial center at the Helsinki University, Finland. All authors contributed to conception of and designing the trial. *IR* drafted the manuscript. *JB* provided cost-effectiveness expertise in clinical trial design. *LDK* provided statistical expertise and will conduct the primary statistical analysis. KSF drafted the sections for the microbiota and fMRI ancillary studies. *MCAK* and *LML* drafted the section for the persuasive technology study. *HAHW*, *SKJ*, *MHS*, *RN*, *IAB*, *MRO*, *KHP*, *RV* and *MV* critically reviewed the manuscript. All authors approved the final version.

Funding

Funding for this research is provided by EU Horizon 2020 PROMISS Project ‘Prevention Of Malnutrition In Senior Subjects in the EU’, Grant agreement no. 678732. Funding sponsors did not participate in the study design, collection, management, analysis and interpretation of data; or writing of the manuscript. They did not participate in the decision to submit the report for publication, nor had ultimate authority over any of these activities.

Competing interests

The authors declare that they have no competing interests.

Protein enriched food products

Protein enriched food products are provided by Kellogg and Fonterra. Costs for these products are also funded through the EU Horizon 2020 PROMISS grant.

For peer review only

Consent for publication

Not applicable. Personal data were not identifiable during the analysis.

Availability of data and materials

Datasets from this research will be stored at the repository of the Vrije Universiteit Amsterdam, the Netherlands and potentially available for other researchers after submitting a research proposal.

Acknowledgements

We acknowlegde the members of the PROMISS trial group and we thank the study participants. We thank Jan de Vries for his ethical advice, and Martin den Heijer and Kaisu Pitkälä for their medical advice.

For peer review only

For peer review only - http://bmjopen.bmj.com/site/about/guidelines.xhtml

Figure legends

Figure 1. Study timeline of the PROMISS trial

For peer review only

For peer review only - http://bmjopen.bmj.com/site/about/guidelines.xhtml

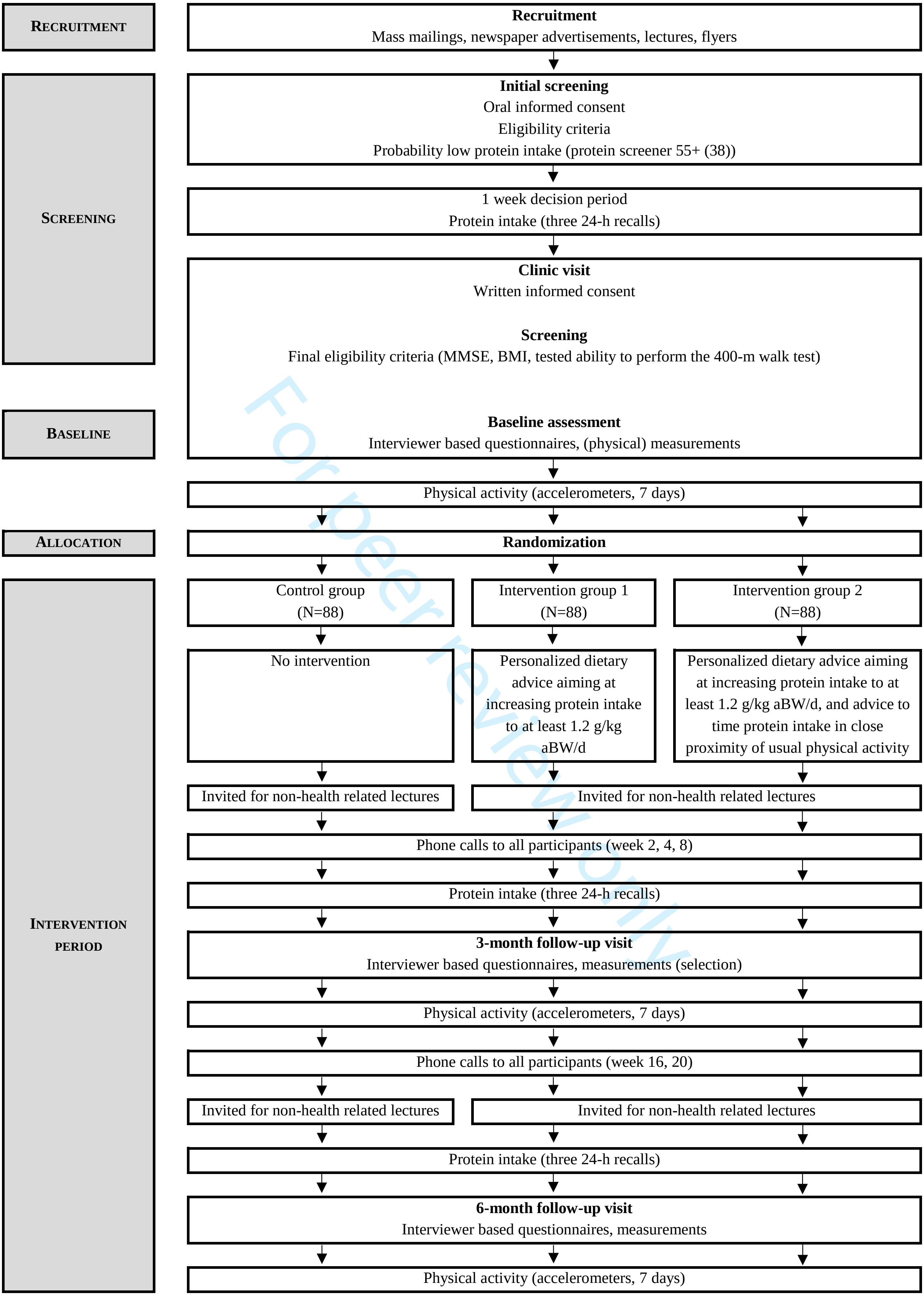

For peer review only

Appendix

Appendix I

PROMISS project coordination, Vrije Universiteit Amsterdam, Department of Health Sciences, the Netherlands

Prof. Marjolein Visser, PhD – Principle investigator of the PROMISS project

Prof. Ingeborg A Brouwer, PhD – Project manager of the PROMISS project

Margreet Olthof, PhD – Financial manager of the PROMISS project

Rachel Vijlbrief – Assistant project manager of the PROMISS project

Trial sites

Vrije Universiteit Amsterdam, the Netherlands

Hanneke Wijnhoven, PhD - local principal investigator

Nanouk Bakker Schut – research intern

Judith Bosmans, PhD – researcher

Mariska Bout – dietician and research assistant

Ingeborg Brouwer, PhD – researcher

Nona Kerremans – research intern

Lothar Kuijper, PhD – researcher

Margreet Olthof, PhD – researcher

Ilse Reinders, PhD – local co-principal investigator

Marjon Veeke – dietician and research assistant

Rachel Vijlbrief – researcher

Marjolein Visser, PhD – researcher

Merel Vrijmoeth – dietician and research assistant

For peer review only

Laura Winkens – researcher

University of Helsinki

Merja Suominen, PhD – local principal investigator

Kirsi Ali-Kovero – research assistant

Johannes Anttila – research intern

Aliisa Hyvönen – dietician and research assistant

Henriikka Jussila – research intern

Satu Jyväkorpi, PhD – local co-principal investigator

Riikka Niskanen – dietician and research assistant

Anna-Maria Piipponen – research intern and research assistant

Kaisu Pitkälä, PhD – researcher

Heli Salmenius-Suominen – researcher

Ancillary studies

Persuasive technology study

Michel Klein, PhD – principal investigator of the persuasive technology study, *Vrije Universiteit Amsterdam, the Netherlands*

Laura van der Lubbe – researcher, *Vrije Universiteit Amsterdam, the Netherlands*

Microbiota study and fMRI study

Fredrik Bäckhed, MD, PhD – researcher, *University of Gothenburg, Gothenburg, Sweden and University of Copenhagen, Copenhagen, Denmark*

Kristien Fluitman, MD – researcher, *Amsterdam UMC, location VUmc, Amsterdam, the Netherlands*

For peer review only

Richard Ijzerman, MD, PhD – researcher, *Amsterdam UMC, location VUmc, Amsterdam, the Netherlands*

Bart Keijser, PhD – researcher, *TNO earth, Zeist, the Netherlands and Academic Center for Dentistry Amsterdam, the Netherlands*

Max Nieuwdorp, MD, PhD – principal investigator of the microbiota study and fMRI study, *Amsterdam UMC, location AMC and location VUmc, Amsterdam, the Netherlands*

For peer review only

Toestemmingsformulier proefpersoon

Effectiviteit van het verhogen van eiwitinname op fysiek functioneren

- Ik heb de informatiebrief gelezen. Ook kon ik vragen stellen. Mijn vragen zijn voldoende beantwoord. Ik had genoeg tijd om te beslissen of ik meedoe.
- Ik weet dat meedoen vrijwillig is. Ook weet ik dat ik op ieder moment kan beslissen om toch niet mee te doen of te stoppen met het onderzoek. Daarvoor hoef ik geen reden te geven.
- Ik geef toestemming voor het informeren van mijn huisarts dat ik meedoe aan dit onderzoek.
- Ik geef toestemming voor het informeren van mijn huisarts over eventuele afwijkingen die tijdens het onderzoek gevonden worden.
- Ik geef toestemming voor het opvragen van informatie bij mijn huisarts over mijn nierfunctie, mochten er twijfels over de effecten op mijn gezondheid bestaan.
- Ik weet dat sommige mensen mijn gegevens kunnen inzien. Die mensen staan vermeld in deze informatiebrief.
- Ik geef toestemming voor het verzamelen en gebruiken van mijn gegevens op de manier en voor de doelen die in de informatiebrief staan.
- Ik geef toestemming om mijn gegevens op de onderzoekslocatie nog 15 jaar na dit onderzoek te bewaren.
- Ik wil meedoen aan dit onderzoek.

Naam proefpersoon:

Handtekening: Datum : __ / __ / __

Ik verklaar dat ik deze proefpersoon volledig heb geïnformeerd over het genoemde onderzoek.

Als er tijdens het onderzoek informatie bekend wordt die de toestemming van de proefpersoon zou kunnen beïnvloeden, dan breng ik hem/haar daarvan tijdig op de hoogte.

Naam onderzoeker (of diens vertegenwoordiger):

Handtekening: Datum: __ / __ / __

For peer review only - http://bmjopen.bmj.com/site/about/guidelines.xhtml

SPIRIT 2013 Checklist: Recommended items to address in a clinical trial protocol and related documents*

Section/item	ItemNo	Description	Page number, section
**Administrative information**			
Title	1	Descriptive title identifying the study design, population, interventions, and, if applicable, trial acronym	1
Trial registration	2a	Trial identifier and registry name. If not yet registered, name of intended registry	4; end of abstract, 9; study design
	2b	All items from the World Health Organization Trial Registration Data Set	N/A
Protocol version	3	Date and version identifier	4; trial status
Funding	4	Sources and types of financial, material, and other support	49; funding
Roles and responsibilities	5a	Names, affiliations, and roles of protocol contributors	1, 49; author contributions
	5b	Name and contact information for the trial sponsor	-
	5c	Role of study sponsor and funders, if any, in study design; collection, management, analysis, and interpretation of data; writing of the report; and the decision to submit the report for publication, including whether they will have ultimate authority over any of these activities	49; funding
	5d	Composition, roles, and responsibilities of the coordinating centre, steering committee, endpoint adjudication committee, data management team, and other individuals or groups overseeing the trial, if applicable (see Item 21a for data monitoring committee)	Appendix II
**Introduction**			

For peer review only - http://bmjopen.bmj.com/site/about/guidelines.xhtml

Background and rationale	6a	Description of research question and justification for undertaking the trial, including summary of relevant studies (published and unpublished) examining benefits and harms for each intervention	6, 7, 8, 9
	6b	Explanation for choice of comparators	8
Objectives	7	Specific objectives or hypotheses	8; second paragraph
Trial design	8	Description of trial design including type of trial (eg, parallel group, crossover, factorial, single group), allocation ratio, and framework (eg, superiority, equivalence, noninferiority, exploratory)	9; study design
**Methods: Participants, interventions, and outcomes**			
Study setting	9	Description of study settings (eg, community clinic, academic hospital) and list of countries where data will be collected. Reference to where list of study sites can be obtained	9-11; eligibility criteria, 12-14; recruitment and screening
Eligibility criteria	10	Inclusion and exclusion criteria for participants. If applicable, eligibility criteria for study centres and individuals who will perform the interventions (eg, surgeons, psychotherapists)	9-11; eligibility criteria
Interventions	11a	Interventions for each group with sufficient detail to allow replication, including how and when they will be administered	16, 21-22; intervention
	11b	Criteria for discontinuing or modifying allocated interventions for a given trial participant (eg, drug dose change in response to harms, participant request, or improving/worsening disease)	-
	11c	Strategies to improve adherence to intervention protocols, and any procedures for monitoring adherence (eg, drug tablet return, laboratory tests)	22; compliance
	11d	Relevant concomitant care and interventions that are permitted or prohibited during the trial	-
Outcomes	12	Primary, secondary, and other outcomes, including the specific measurement variable (eg, systolic blood pressure), analysis metric (eg, change from baseline, final value, time to event), method of aggregation (eg, median, proportion), and time point for each outcome. Explanation of the clinical relevance of chosen efficacy and harm outcomes is strongly recommended	23; primary outcome, 23-26; secondary outcomes, 26-27; other measures

For peer review only - http://bmjopen.bmj.com/site/about/guidelines.xhtml

Participant timeline	13	Time schedule of enrolment, interventions (including any run-ins and washouts), assessments, and visits for participants. A schematic diagram is highly recommended (see Figure)	15-16; study timeline, Figure 1
Sample size	14	Estimated number of participants needed to achieve study objectives and how it was determined, including clinical and statistical assumptions supporting any sample size calculations	27; sample size
Recruitment	15	Strategies for achieving adequate participant enrolment to reach target sample size	12-14; recruitment and screening

Methods: Assignment of interventions (for controlled trials)

Allocation:

Sequence generation	16a	Method of generating the allocation sequence (eg, computer-generated random numbers), and list of any factors for stratification. To reduce predictability of a random sequence, details of any planned restriction (eg, blocking) should be provided in a separate document that is unavailable to those who enrol participants or assign interventions	14; Randomization, allocation and masking
Allocation concealment mechanism	16b	Mechanism of implementing the allocation sequence (eg, central telephone; sequentially numbered, opaque, sealed envelopes), describing any steps to conceal the sequence until interventions are assigned	-
Implementation	16c	Who will generate the allocation sequence, who will enrol participants, and who will assign participants to interventions	15; second paragraph of study time line
Blinding (masking)	17a	Who will be blinded after assignment to interventions (eg, trial participants, care providers, outcome assessors, data analysts), and how	14; Randomization, allocation and masking, 27; statistical analyses plan
	17b	If blinded, circumstances under which unblinding is permissible, and procedure for revealing a participant's allocated intervention during the trial	-

Methods: Data collection, management, and analysis

Data collection methods	18a	Plans for assessment and collection of outcome, baseline, and other trial data, including any related processes to promote data quality (eg, duplicate measurements, training of assessors) and a description of study instruments (eg, questionnaires, laboratory tests) along with their reliability and validity, if known. Reference to where data collection forms can be found, if not in the protocol	15-16; study timeline, 22-23, intervention fidelity, 23; primary outcome, 23-26; secondary outcomes, 26-27; other measures
	18b	Plans to promote participant retention and complete follow-up, including list of any outcome data to be collected for participants who discontinue or deviate from intervention protocols	-
Data management	19	Plans for data entry, coding, security, and storage, including any related processes to promote data quality (eg, double data entry; range checks for data values). Reference to where details of data management procedures can be found, if not in the protocol	29-30; Data quality assurance and data management
Statistical methods	20a	Statistical methods for analysing primary and secondary outcomes. Reference to where other details of the statistical analysis plan can be found, if not in the protocol	27-29; Statistical analyses plan
	20b	Methods for any additional analyses (eg, subgroup and adjusted analyses)	27-29; Statistical analyses plan
	20c	Definition of analysis population relating to protocol non-adherence (eg, as randomised analysis), and any statistical methods to handle missing data (eg, multiple imputation)	27-29; Statistical analyses plan
**Methods: Monitoring**			
Data monitoring	21a	Composition of data monitoring committee (DMC); summary of its role and reporting structure; statement of whether it is independent from the sponsor and competing interests; and reference to where further details about its charter can be found, if not in the protocol. Alternatively, an explanation of why a DMC is not needed	-
	21b	Description of any interim analyses and stopping guidelines, including who will have access to these interim results and make the final decision to terminate the trial	-

For peer review only - http://bmjopen.bmj.com/site/about/guidelines.xhtml

Harms	22	Plans for collecting, assessing, reporting, and managing solicited and spontaneously reported adverse events and other unintended effects of trial interventions or trial conduct	29; Participants' safety
Auditing	23	Frequency and procedures for auditing trial conduct, if any, and whether the process will be independent from investigators and the sponsor	-
**Ethics and dissemination**			
Research ethics approval	24	Plans for seeking research ethics committee/institutional review board (REC/IRB) approval	36; Ethics and dissemination
Protocol amendments	25	Plans for communicating important protocol modifications (eg, changes to eligibility criteria, outcomes, analyses) to relevant parties (eg, investigators, REC/IRBs, trial participants, trial registries, journals, regulators)	-
Consent or assent	26a	Who will obtain informed consent or assent from potential trial participants or authorised surrogates, and how (see Item 32)	12-14; recruitment and screening, 15-16; study timeline, 36; Ethics and dissemination
	26b	Additional consent provisions for collection and use of participant data and biological specimens in ancillary studies, if applicable	31-32; Persuasive technology study, 32-33; Microbiota study, 33-34; fMRI study.
Confidentiality	27	How personal information about potential and enrolled participants will be collected, shared, and maintained in order to protect confidentiality before, during, and after the trial	29-30; Data quality assurance and data management<br><br>50; Consent for publication
Declaration of interests	28	Financial and other competing interests for principal investigators for the overall trial and each study site	49; Competing interests
Access to data	29	Statement of who will have access to the final trial dataset, and disclosure of contractual agreements that limit such access for investigators	50; Availability of data and materials

For peer review only

Ancillary and post-trial care	30	Provisions, if any, for ancillary and post-trial care, and for compensation to those who suffer harm from trial participation	N/A
Dissemination policy	31a	Plans for investigators and sponsor to communicate trial results to participants, healthcare professionals, the public, and other relevant groups (eg, via publication, reporting in results databases, or other data sharing arrangements), including any publication restrictions	-
	31b	Authorship eligibility guidelines and any intended use of professional writers	-
	31c	Plans, if any, for granting public access to the full protocol, participant-level dataset, and statistical code	-
**Appendices**			
Informed consent materials	32	Model consent form and other related documentation given to participants and authorised surrogates	Appendix I
Biological specimens	33	Plans for collection, laboratory evaluation, and storage of biological specimens for genetic or molecular analysis in the current trial and for future use in ancillary studies, if applicable	N/A

*It is strongly recommended that this checklist be read in conjunction with the SPIRIT 2013 Explanation & Elaboration for important clarification on the items. Amendments to the protocol should be tracked and dated. The SPIRIT checklist is copyrighted by the SPIRIT Group under the Creative Commons "Attribution-NonCommercial-NoDerivs 3.0 Unported" license.

For peer review only

For peer review only - http://bmjopen.bmj.com/site/about/guidelines.xhtml
